# Endothelial Cells Mediated by STING Regulate Oligodendrogenesis and Myelination During Brain Development

**DOI:** 10.1002/advs.202308508

**Published:** 2024-08-13

**Authors:** Wenwen Wang, Yanyan Wang, Libo Su, Mengtian Zhang, Tianyu Zhang, Jinyue Zhao, Hongyan Ma, Dongming Zhang, Fen Ji, Ryan Dingli Jiao, Hong Li, Yuming Xu, Lei Chen, Jianwei Jiao

**Affiliations:** ^1^ Key Laboratory of Organ Regeneration and Reconstruction Chinese Academy of Science Beijing 100101 China; ^2^ School of Life Sciences University of Science and Technology of China Hefei 230026 China; ^3^ University of Chinese Academy of Sciences Beijing 100049 China; ^4^ Beijing Royal School Beijing 102209 China; ^5^ Department of Neurology The First Affiliated Hospital of Zhengzhou University Zhengzhou 450000 China; ^6^ Department of Neurology West China Hospital Sichuan University Chengdu 610041 China; ^7^ Co‐Innovation Center of Neuroregeneration Nantong University Nantong 226001 China; ^8^ Beijing Institute for Stem Cell and Regenerative Medicine Institute for Stem Cell and Regeneration Chinese Academy of Sciences Beijing 100101 China

**Keywords:** Brain development, Endothelial cells, Oligodendrogenesis, Sting

## Abstract

Oligodendrocyte precursor cells (OPCs) migrate extensively using blood vessels as physical scaffolds in the developing central nervous system. Although the association of OPCs with the vasculature is critical for migration, the regulatory mechanisms important for OPCs proliferative and oligodendrocyte development are unknown. Here, a correlation is demonstrated between the developing vasculature and OPCs response during brain development. Deletion of endothelial stimulator of interferon genes (STING) disrupts angiogenesis by inhibiting farnesyl‐diphosphate farnesyltransferase 1 (FDFT1) and thereby reducing cholesterol synthesis. Furthermore, the perturbation of metabolic homeostasis in endothelial cells increases interleukin 17D production which mediates the signal transduction from endothelial cells to OPCs, which inhibits oligodendrocyte development and myelination and causes behavioral abnormalities in adult mice. Overall, these findings indicate how the endothelial STING maintains metabolic homeostasis and contributes to oligodendrocyte precursor cells response in the developing neocortex.

## Graphical abstract

Oligodendrocytes are differentiated from oligodendrocyte progenitors and are the myelinating cells of the central nervous system. In this study, it is demonstrated a correlation between the developing vasculature and OPCs proliferative and oligodendrocyte development. This work provides new insights into how the endothelial STING maintains metabolic homeostasis and contributes to oligodendrocyte precursor cells response in the developing neocortex.

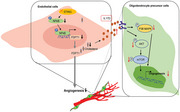

## 1 Introduction

Oligodendrocytes are differentiated from oligodendrocyte progenitors and are the myelinating cells of the central nervous system.^[^ ^1^, ^2^ ^]^ Oligodendrogenesis at the developmental stage is associated with a group of disorders known as demyelinating diseases, such as periventricular leukomalacia and multiple sclerosis.^[^ ^3^ ^]^ Therefore, understanding the regulatory mechanism of the proliferation and differentiation of OPCs is fundamental to treating these diseases.

In mouse brains, oligodendrocyte precursor cells (OPCs) appear in three waves; they first appear in the medial ganglion bulge (MGE) and the anterior dorsal medial region (AEP) from the ventral forebrain at embryonic day (E) 12, followed by the lateral and/or caudal ganglion bulge (LGE and/or CGE, respectively), and subsequently enter the brain after E16. The third wave originates from the latter wave in the postnatal cortex.^[^ ^4^ ^]^ OPCs are associated with blood vessels distributed throughout the central nervous system (CNS), which involves direct contact with the surface of endothelial cells (ECs). Blood vessels play a guiding role in the process of OPCs migration, indicating that OPCs are closely related to the developing vasculature.^[^ ^5^ ^]^ However, the role of the developing vascular system in regulating oligodendrogenesis is largely unexplored.

The endoplasmic reticulum (ER)‐resident stimulator of interferon genes (STING) plays important roles in inflammation and pathogen defense by regulating type I interferon signaling.^[^ ^6^, ^7^, ^8^, ^9^ ^]^ In addition to these functions, recent studies have highlighted the role of STING in physiological states, such as metabolism and protein synthesis.^[^ ^10^, ^11^ ^]^ Activation of STING leads to the inflammation of blood vessels, known as STING‐associated vasculopathy with onset in infancy (SAVI),^[^ ^12^ ^]^ and notably, the STING signal may be important in vascular biology. Some studies indicate that the STING protein is expressed in ECs of the tumor tissue, and STING activation can reprogram tumor vasculatures and synergizes with vascular endothelial growth factor receptor 2 (VEGFR2) blockade.^[^ ^13^ ^]^ However, the role of STING signaling in ECs during brain development has never been investigated.

Here, we found that the loss of endothelial STING reduces angiogenesis and affects oligodendrogenesis during brain development. Mechanistically, STING inhibits cholesterol synthesis by regulating FDFT1 expression through NF‐𝜅B phosphorylation to disrupt angiogenesis. Additionally, IL17D, which is driven by endothelial cells, binds to the CD93 receptor to regulate OPCs. Overall, our data provide a new insight into the roles of STING signaling in ECs during brain development.

## 2 Results

### 2.1 STING Expression is Closely Correlated with Endothelial Cells during Brain Development

**Figure 1:**
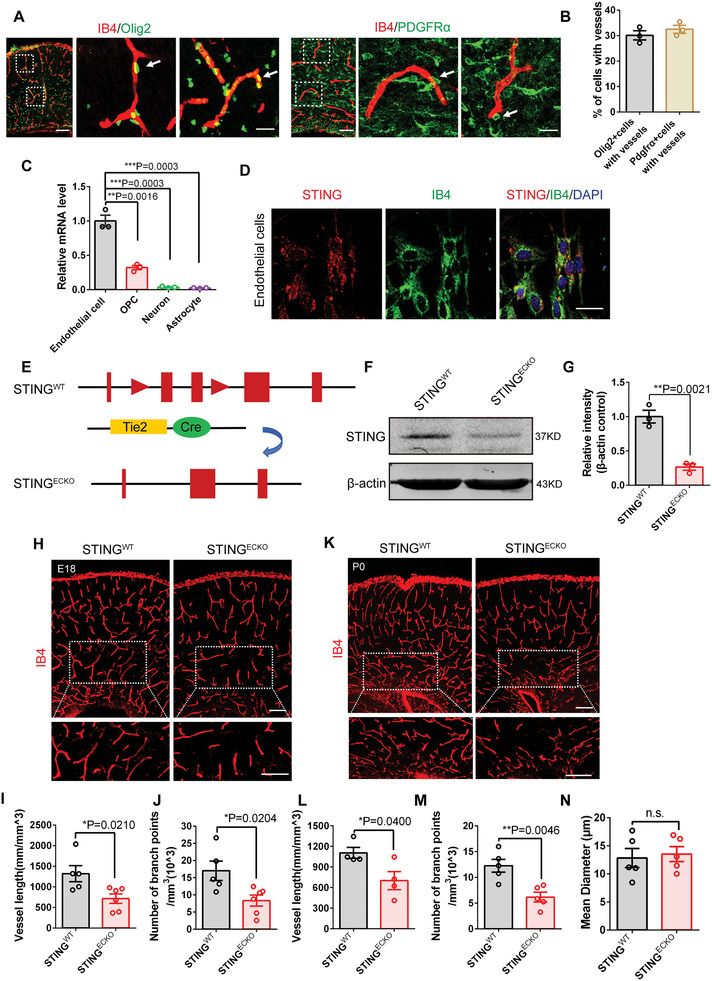
STING expression closely correlates with endothelial cells and deletion of endothelial STING reduces angiogenesis. A) Oligodendrocyte precursor cells and endothelial cells interactions in E18 mouse forebrain coronal section. Confocal immunofluorescence image of IB4 and Olig2 showed that blood vessels were located near OPCs (white arrowheads). Confocal immunofluorescence image of IB4 and PDGFRα showed that blood vessels were located near OPCs (white arrowheads). The right is higher magnification images. Scale bars, 100 µm (left), 20 µm (right). B) Quantification of OPC association with vessels at E18 (707 cells measured, n = 3 mice). C) RT‐PCR was performed to detect the mRNA levels of STING in endothelial cells, OPC, Neuron, and Astrocyte. ^* *^ *P* < 0.01, ^* * *^ *P* < 0.001 (mean ± SEM, n = 3 mice each group). D) Confocal immunofluorescence image of STING and IB4 in endothelial cells showing the localization of STING in ECs. Scale bar, 20 µm. E) Schematic of the endothelial STING conditional knockout mice construction strategy. Tie2‐cre was endothelial cells‐specific recombinase. F) Western blot analysis of the expression levels of STING in the *STING^WT^* and *STING^ECKO^* isolated brain endothelial cells at P0. β‐actin was detected as a loading control. G) Statistics of relative intensity of STING showing the decreased expression of STING in isolated brain endothelial cells of *STING^ECKO^* mice. ^* *^ *P* < 0.01 (mean ± SEM, unpaired two‐tailed Student's *t*‐test, n = 3 mice each group). H) Confocal immunofluorescence image of IB4 at E18 in the *STING^WT^* and *STING^ECKO^* cortical sections. The down is higher magnification images. Scale bars, 100 µm (up), 50 µm (down). I,J) Quantification of the vessel length (I) and branch points (J), and showing a decreased vessel length and branch points in *STING^ECKO^* cortical sections. ^*^ *P* < 0.05 (mean ± SEM, unpaired two‐tailed Student's *t*‐test, *STING^WT^* n = 5 mice; *STING^ECKO^* n = 6 mice). K) Confocal immunofluorescence image of IB4 at P0 in the *STING^WT^* and *STING^ECKO^* cortical sections. The down is higher magnification images. Scale bars, 100 µm (up), 50 µm (down). L–N) Quantification of the vessel length (L), branch points (M) and mean diameter (N) and showing a decreased vessel length and branch points in *STING^ECKO^* cortical sections. n.s., not significant, ^*^ *P* < 0.05, ^* *^ *P* < 0.01 (mean ± SEM, unpaired two‐tailed Student's *t*‐test, L) n = 4 mice each group, M) n = 5 mice each group, K) n = 5 mice each). Data are represented as means ± SEM. unpaired two‐tailed Student's *t‐*test; At least three biological replicates are shown. n.s., not significant, ^*^ *P* < 0.05, ^* *^ *P* < 0.01, ^* * *^ *P* < 0.001.

Previous studies have demonstrated that OPCs are correlated with perivascular migration.^[^ ^5^, ^14^ ^]^ We first investigated the possibility of the physical interaction of OPCs with ECs during brain development. Immunofluorescence staining showed that blood vessels, visualized using biotinylated isolectin B4 (IB4), were physically co‐located with Olig2^+^ (the oligodendrocyte‐lineage marker) cells and PDGFRα^+^ (a marker of OPCs) cells (**Figure** **1**A,B). These results suggested that the developing blood vessels were closely correlated with OPCs. Previous studies have indicated that pharmacological inhibition of STING protected mice from permeability defects and proinflammatory endothelial changes during sepsis,^[^ ^15^ ^]^ indicating that STING might have important roles in ECs. To investigate the role of STING in the vascular and neuron systems of the developing brain, we first examined the expression of STING in ECs, OPCs, neurons, and astrocytes, and found that STING expression in ECs was the highest (Figure 1C). Moreover, cellular immunostaining also demonstrated that STING was expressed in primary ECs (Figure 1D). Additionally, we evaluated STING expression in different stages of brain EC development, and found that it increased gradually with brain development (Figure S1A, Supporting Information). Overall, the colocalization with IB4 suggests that STING might be involved in ECs development.

### 2.2 Loss of Endothelial STING Reduces Angiogenesis during Brain Development

To examine the function of endothelial STING in cortex development, endothelial conditional knockout mice (*STING^ECKO^*) were developed by crossing *STING^flox/flox^* mice with a Tie2‐Cre driver that targets the ECs lineage (Figure 1E). In order to visualize ECs populations, Tie2‐cre were crossed with Rosa26tdTomato mice to generate Tek‐cre; Rosa26tdTomato^+^mice.The result showed that tdTomato were co‐located with IB4‐labeled blood vessels (Figure S1B, Supporting Information). Subsequently, western blot and cell immunofluorescent analysis showed that STING expression was down in the brain ECs of *STING^ECKO^* (Figure 1F,G; Figure S1C–E, Supporting Information). We assessed whether cerebral vascular morphology changed after STING knockout in ECs. IB4 immunostaining showed that the vessel length, number of branch points, and average diameter of the blood vessels decreased compared with those of *STING^WT^* littermates at E18 and postnatal day (P) 0 (Figure 1H–N). These results suggest that the loss of endothelial STING affects the vascular morphology of the brain. We then conducted BrdU and Ki67 immunostaining in sorted primary brain ECs from *STING^WT^* and *STING^ECKO^* mice, and found that the percentages of ECs in BrdU^+^ and Ki67^+^ reduced after the deletion of endothelial STING (Figure S1F–H, Supporting Information). Moreover, other blood vessel markers, namely, CD31 and CDH5, were used to label growing vessels in the cerebral cortex. Reverse transcription‐polymerase chain reaction (RT‐PCR) analysis showed that the expression levels of STING, CD31 and CDH5 decreased in brain ECs from *STING^ECKO^* mice (Figure S1I, Supporting Information). Cyclic guanosine monophosphate (GMP)‐adenosine monophosphate (AMP) (cGAMP) synthase (cGAS) is a cytosolic DNA sensor, which generates cGAMP, namely, a diffusible cyclic dinucleotide that activates the adaptor STING.^[^ ^16^, ^17^ ^]^ Knockdown of cGAS in ECs reduced the expression levels of blood vessel markers CD31 and CDH5 (Figure S1J, Supporting Information). We found that cGAMP increased CD31 expression after treating endothelial cells with cGAMP for one day, while there were no significant changes in the *STING^ECKO^* mice (Figure S1K,L, Supporting Information). These results demonstrate that STING signaling is required for angiogenesis during brain development.

The blood‐brain barrier (BBB) is well characterized, comprising microvascular ECs with tight junctions.^[^ ^18^ ^]^ To explore the role of endothelial STING during BBB development, we first assessed the integrity of endothelial tight junctions in *STING^ECKO^* mice. We found that tight junction proteins, namely, claudin‐5 and ZO‐1, showed no changes after the loss of endothelial STING (Figure S2A–D, Supporting Information). We then evaluated the BBB integrity using the fluorescent tracer AlexaFluor 555 cadaverine (Cad‐A555), and found no leakage of cadaverine from blood vessels in *STING^WT^* and *STING^ECKO^* mice (Figure S2E, Supporting Information). Recent studies suggest that the basement membrane plays important roles in the vascular barrier.^[^ ^19^ ^]^ Collagen IV (a marker of the EC basement membrane) immunostaining showed no significant differences between *STING^WT^* and *STING^ECKO^* mice (Figure S2F,G, Supporting Information). Overall, these results indicate that BBB integrity is maintained in *STING^ECKO^* animals.

### 2.3 Loss of Endothelial STING Inhibits OPC Proliferation during Brain Development

**Figure 2:**
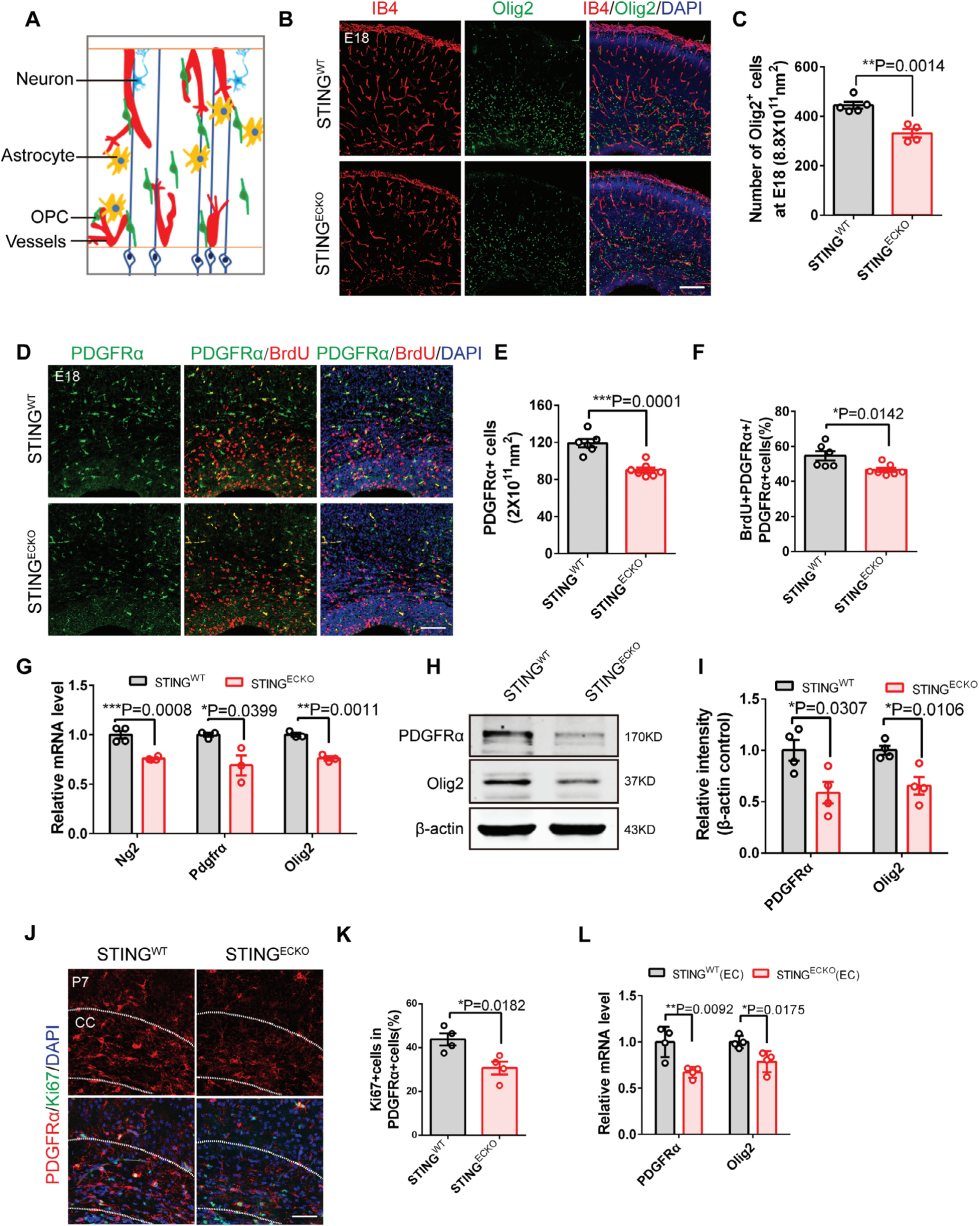
Loss of endothelial STING inhibits oligodendrocyte progenitor cells proliferation during brain development. A) Mode pattern of OPCs, neurons, and astrocytes interactions with developing vasculature in the forebrain. B) Confocal immunofluorescence image of IB4^+^ and Olig2^+^ cells at E18 in the *STING^WT^* and *STING^ECKO^* cortical sections Scale bar, 200 µm. C) Quantification of the number of Olig2^+^ cells showing the decreased Olig2^+^ cells in *STING^ECKO^* mice. ^* *^ *P* < 0.01 (mean ± SEM, unpaired two‐tailed Student's *t*‐test, *STING^WT^* n = 5 mice; *STING^ECKO^* n = 4 mice). D) Confocal immunofluorescence image of BrdU^+^ PDGFRα^+^ cells at E18 in the *STING^WT^* and *STING^ECKO^* cortical sections Scale bar, 100 µm. E) Quantification showing the decreased number of PDGFRα^+^ oligodendrocyte precursor cells in *STING^ECKO^* mice. ^* * *^ *P* < 0.001 (mean ± SEM, unpaired two‐tailed Student's t test, *STING^WT^* n = 6 mice; *STING^ECKO^* n = 7 mice). F) Quantification showing the decreased number of BrdU^+^ PDGFRα^+^ oligodendrocyte precursor cells in *STING^ECKO^* mice. ^*^ *P* < 0.05 (mean ± SEM, unpaired two‐tailed Student's t test, *STING^WT^* n = 6 mice; *STING^ECKO^* n = 7 mice). G) RT‐PCR analysis of the mRNA expression level of Ng2, Pdgfrα, and Olig2(oligodendrocyte precursor cells markers) at E18 in the *STING^WT^* and *STING^ECKO^* brain. ^*^ *P* < 0.05, ^* *^ *P* < 0.01, ^* * *^ *P* < 0.001 (mean ± SEM, unpaired two‐tailed Student's *t*‐test, Ng2: *STING^WT^* n = 4 each group; Pdgfrα and Olig2: n = 3 mice each group). H) Western blot analysis of the expression levels of PDGFRα and OLIG2 at E18 in the *STING^WT^* and *STING^ECKO^* brain. β‐actin was detected as a loading control. I) Statistics of relative intensity of PDGFRα and OLIG2 showing decreased the expression of PDGFRα^+^ and OLIG2^+^cells in P0 *STING^ECKO^* mice. ^*^ *P* < 0.05 (mean ± SEM, unpaired two‐tailed Student's *t*‐test, n = 4 mice each group). J) Confocal immunofluorescence image of Ki67^+^ PDGFRα^+^ cells at P7 in the *STING^WT^* and *STING^ECKO^* corpus callosum sections Scale bar, 50 µm. K) Quantification of the percent of Ki67^+^cells among PDGFRα^+^ cells showing loss of endothelial STING affected the proliferation of OPCs. ^*^ *P* < 0.05(mean ± SEM, unpaired two‐tailed Student's *t*‐test, n = 4 mice each group). L) RT‐PCR analysis of the mRNA expression level of PDGFRα and Olig2 in a co‐culture system. ^*^ *P* < 0.05, ^* *^ *P* < 0.01 (mean ± SEM, unpaired two‐tailed Student's *t*‐test, n = 4 mice each group). Data are represented as means ± SEM. unpaired two‐tailed Student's *t‐*test; At least three biological replicates are shown. ^*^ *P* < 0.05. ^* *^ *P* < 0.01, ^* * *^ *P* < 0.001.

As OPCs interact with ECs in the developing brain (**Figure** **2**A), we examined whether abnormal vascular development affects OPCs behaviors. Previous studies have shown that blood vessels support the migration of OPCs.^[^ ^5^ ^]^ We did not observe the gathering of OPCs on blood vessels, indicating that the OPCs migration was not affected (Figure 2B; Figure S3A,B, Supporting Information). Notably, immunofluorescence staining confirmed that the numbers of Olig2^+^ cells and PDGFRα^+^OPCs decreased in the brain cortex of *STING^ECKO^* mice (Figure 2B–E). Additionally, BrdU^+^ PDGFRα^+^ OPCs also decreased after the removal of STING in ECs, suggesting that endothelial STING affects the proliferation of OPCs (Figure 2F). Furthermore, we found that the expression levels of NG2, PDGFRα, and OLIG2 were significantly reduced in the brain cortex of *STING^ECKO^* mice (Figure 2G). And the protein expression levels of PDGFRα and Olig2 were significantly reduced in the brain of *STING^ECKO^* mice (Figure 2H,I). Subsequently, PDGFRα and Ki67 immunofluorescence staining showed that OPCs proliferation significantly decreased in *STING^ECKO^* mice at P7 (Figure 2J,K). And the expression levels of PDGFRα and OLIG2 were significantly reduced in the brain of *STING^ECKO^* mice (Figure 2L). Overall, these results indicate that loss of endothelial STING inhibits OPCs proliferation during brain development.

Blood vessel growth occurs simultaneously as neural progenitor cells sequentially give rise to neurons and astrocytes during brain development.^[^ ^20^ ^]^ So, we first explored whether neurons and astrocytes were affected by the deletion of STING in ECs. Immunofluorescence staining showed that the numbers of NeuN^+^ neurons and GFAP^+^ astrocytes were comparable between those of *STING^WT^* and *STING^ECKO^* mice (Figure S3C–F). Additionally, the expression levels of TUJ1 (neuron marker) and ALDH1L1 (astrocyte marker) showed no significant differences between *STING^WT^* and *STING^ECKO^* mice (Figure S3G,H, Supporting Information). These results suggested that the numbers of neurons and astrocytes were not altered when endothelial STING was lost. To determine whether the observed reduction in oligodendrocyte numbers owing to the loss of endothelial STING results from the death of oligodendrocyte‐lineage cells, we conducted TUNEL (an apoptosis marker) immunofluorescent staining. The results showed that the number of TUNEL was not affected after the loss of endothelial STING, suggesting that endothelial STING promotes the proliferation of OPCs in the developing brain and that this does not occur by inhibiting OPCs apoptosis (Figure S3I,J, Supporting Information).

### 2.4 Deletion of Endothelial STING Alters Oligodendrocytes and Myelination in the Developing Brain

**Figure 3:**
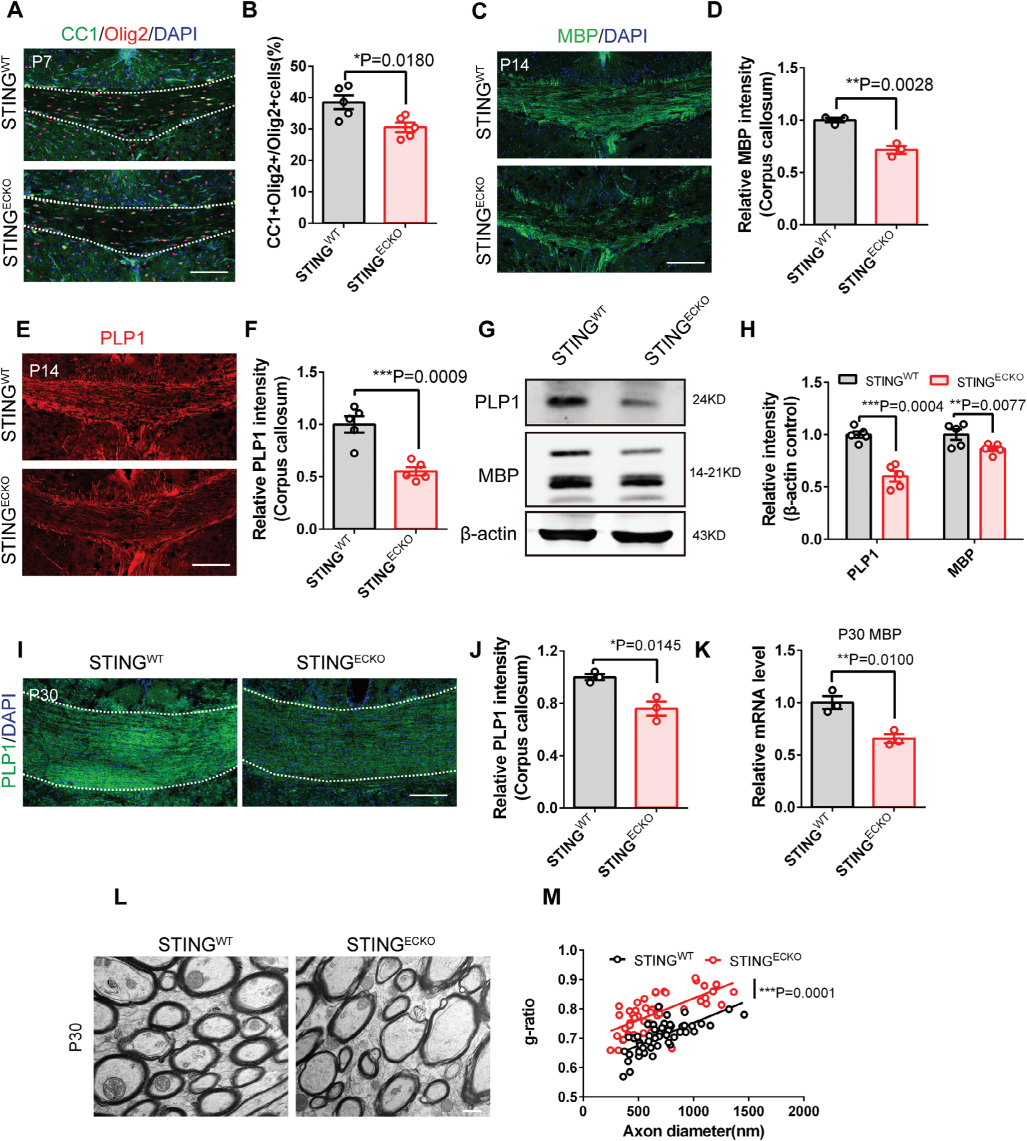
Deletion of endothelial STING causes defects in oligodendrocytes and myelination in the developing brain. A) Confocal immunofluorescence image of CC1^+^ Olig2^+^ cells at P7 in the *STING^WT^* and *STING^ECKO^* corpus callosum. Scale bar, 100 µm. B) Quantification of the percent of CC1^+^ Olig2^+^ cells showing the decreased oligodendrocyte in the *STING^WT^* and *STING^ECKO^* corpus callosum. ^*^ *P* < 0.05 (mean ± SEM, unpaired two‐tailed Student's *t* test, n = 5 mice each group). C) Confocal immunofluorescence image of MBP^+^ cells at P14 in the *STING^WT^* and *STING^ECKO^* corpus callosum. Scale bar, 100 µm. D) Relative quantification of MBP fluorescence intensity in the *STING^WT^* and *STING^ECKO^* corpus callosum. ^* *^ *P* < 0.01 (mean ± SEM, unpaired two‐tailed Student's *t*‐test, n = 3 mice each group). E) Confocal immunofluorescence image of PLP1^+^ cells at P14 in the *STING^WT^* and *STING^ECKO^* corpus callosum. Scale bar, 100 µm. F) Relative quantification of PLP1 fluorescence intensity in the *STING^WT^* and *STING^ECKO^* corpus callosum. ^* * *^ *P* < 0.001 (mean ± SEM, unpaired two‐tailed Student's *t*‐test, n = 5 mice each group). G) Western blot analysis of the expression levels of PLP1 and MBP at P14 in the *STING^WT^* and *STING^ECKO^* mice brain. β‐actin was detected as loading control. H) Statistics of relative intensity of PLP1 and MBP showing decreased the expression of PLP1^+^ and MBP^+^ cells in P14 *STING^ECKO^* mice. ^* *^ *P* < 0.01, ^* * *^ *P* < 0.001 (mean ± SEM, unpaired two‐tailed Student's *t*‐test, n = 5 mice each group). I) Confocal immunofluorescence image of PLP1^+^ cells at P30 in the *STING^WT^* and *STING^ECKO^* corpus callosum. Scale bar, 100 µm. J) Relative quantification of PLP1 fluorescence intensity at P30 in the *STING^WT^* and *STING^ECKO^* corpus callosum. ^*^ *P* < 0.05 (mean ± SEM, unpaired two‐tailed Student's *t*‐test, n = 3 mice each group). K) RT‐PCR analysis of the mRNA expression level of MBP at P30 in the *STING^WT^* and *STING^ECKO^* brain. ^* *^ *P* < 0.01 (mean ± SEM, unpaired two‐tailed Student's *t*‐test, n = 3 mice each group). L) Electron microscopic examination of the corpus callosum at P30 from the *STING^WT^* and *STING^ECKO^* mice. Scale bar, 500 nm. M) Scatterplots of the myelin g‐ratios (diameter of axon/diameter of myelinated fiber) in the corpus callosum of P30 from *STING^WT^* and *STING^ECKO^* mice. ^* * *^ *P* < 0.001 (mean ± SEM, simple linear regression, *STING^WT^* n = 56; *STING^ECKO^* n = 50 from 3 independent experiments). Data are represented as means ± SEM. unpaired two‐tailed Student's *t‐*test; At least three biological replicates are shown. ^*^ *P* < 0.05. ^* *^ *P* < 0.01, ^* * *^ *P* < 0.001.

OPCs differentiate into mature oligodendrocytes that form myelin at the neonatal stage.^[^ ^21^ ^]^ Thus, we examined whether the differentiation of oligodendrocytes is altered. We conducted immunofluorescent staining in the mouse brain at P7 and found that the percentage of CC1 ^+^ cells among Olig2 cells were reduced in the corpus callosum of *STING^ECKO^* mice (**Figure** **3**A,B). MBP (Myelin Basic Protein) and PLP1 (Proteolipid Protein 1) are two prominent proteins found in the white matter of the central nervous system (CNS).^[^ ^22^ ^]^ MBP^+^ and PLP1^+^ expression levels were determined by immunofluorescence and western blot analysis. Immunostaining showed that the expression of MBP and PLP1 was markedly decreased in the corpus callosum of *STING^ECKO^* mice at P14 (Figure 3C–F). Additionally, western blot analysis showed that the expression levels of MBP and PLP1 was significantly decreased after the loss of STING in ECs (Figure 3G,H). Furthermore, the ablation of endothelial STING reduced the total number of Olig2^+^ oligodendrocytes in the corpus callosum at P30 (Figure S4A,B, Supporting Information), while the width of the corpus callosum remains unaffected (Figure S4C, Supporting Information). We found that the expression of MBP and PLP1 was also significantly decreased in the corpus callosum and brain cortex of *STING^ECKO^* mice at P30, respectively (Figure 3I,J; Figure S4D,E, Supporting Information). Furthermore, real‐time PCR analysis showed that the expression of MBP significantly decreased in the corpus callosum and cortex of *STING^ECKO^* mice at P30 (Figure 3K). Overall, our data indicated that the loss of STING in ECs perturbed oligodendrocyte development. Oligodendrocytes form myelin sheaths around axons in the central nervous system (CNS).^[^ ^23^ ^]^ Thus, we employed electron microscopy (EM) to examine the ultrastructure of myelin sheaths in the white matter regions of *STING^WT^* and *STING^ECKO^* mice. The results showed that myelin thickness had a notable decrease in *STING^ECKO^* mice at P30 (Figure 3L,M). Based on the analysis of EM images, we found that the myelinated axon diameter of *STING^ECKO^* mice was unaffected compared with that of *STING^WT^* mice (Figure S4F, Supporting Information). Overall, these results indicate that the ablation of endothelial STING perturbed myelination in the CNS.

### 2.5 Deletion of Endothelial STING Leads to Behavioral Defects in Mice

**Figure 4:**
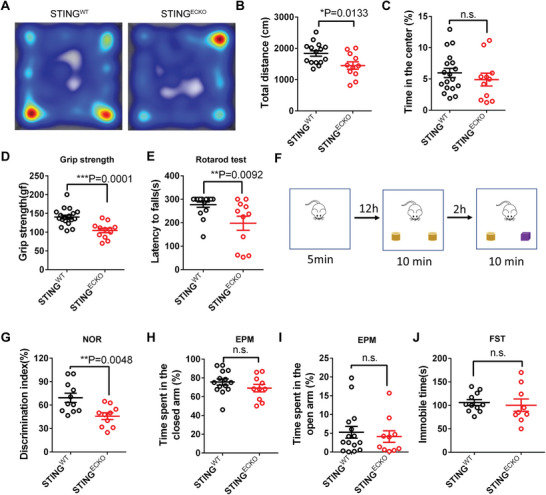
Deletion of endothelial STING leads to behavioral defects in mice. A) Representative tracks of *STING^WT^* and *STING^ECKO^* mice in the open field test. B) Graph shows that the total distance was reduced in *STING^ECKO^* mice in the open field test (*STING^WT^* n = 15 mice; *STING^ECKO^* n = 11 mice). C) Graph shows that time spent in the center was not different between *STING^WT^* and *STING^ECKO^* mice in the open field test (*STING^WT^* n = 18 mice; *STING^ECKO^* n = 11mice). D) The grip strength test shows force of grip strength was reduced in *STING^ECKO^* mice (*STING^WT^* n = 20 mice; *STING^ECKO^* n = 12 mice). E) The rotarod test shows that the forced locomotor activity was significantly different between *STING^WT^* and *STING^ECKO^* mice (*STING^WT^* n = 16 mice; *STING^ECKO^* n = 11mice). F) Schematic illustration of the novel objective recognition (NOR) test. G) Graph shows the percentage of preference for novel object in *STING^WT^* and *STING^ECKO^* mice (*STING^WT^* n = 11mice; *STING^ECKO^* n = 10mice). H) Graph shows time spent on the closed arm between *STING^WT^* and *STING^ECKO^* mice in the elevated‐plus maze test (*STING^WT^* n = 14 mice; *STING^ECKO^* n = 10 mice). I) Graph shows time spent on open arm between *STING^WT^* and *STING^ECKO^* mice in the elevated‐plus maze test (*STING^WT^* n = 15 mice; *STING^ECKO^* n = 10 mice). J) Graph shows that the immobile time was not significantly different between *STING^WT^* and *STING^ECKO^* mice (*STING^WT^* n = 11mice; *STING^ECKO^* n = 9 mice). Data are represented as means ± SEM. unpaired two‐tailed Student's *t‐*test; n.s., not significant, ^*^ *P* < 0.05, ^* *^ *P* < 0.01, ^* * *^ *P* < 0.001.

Myelin dysfunction has a profound effect on locomotion and cognition.^[^ ^21^, ^24^ ^]^ Thus, we evaluated whether abnormal myelination triggered any behavioral defects in *STING^ECKO^* mice. All behavioral experiments were conducted on littermate *STING^WT^* and *STING^ECKO^* mice at ages of 2–4 months. *STING^ECKO^* mice and their littermate *STING^WT^* mice were first tested in the open field. We found that the traveled distance was reduced in *STING^ECKO^* mice, but that there was no statistical difference in the time spent in the center area between *STING^ECKO^* and *STING^WT^* mice (**Figure** **4**A–C). These results suggested changes in the locomotor activity. Subsequently, we conducted a grip strength test to examine their motor capacities. The results showed that the grip strength of *STING^ECKO^* mice significantly decreased compared with that of *STING^WT^* mice (Figure 4D), suggesting locomotor defects in *STING^ECKO^* mice. The rotarod test is a standardized procedure used to assess locomotor learning and balance in animals.^[^ ^25^ ^]^ Consistent with these findings, the rotarod test suggested that the *STING^ECKO^* mice exhibited an increased probability to fall from the spinning rod than littermate *STING^WT^* mice (Figure 4E). These results indicated locomotor and balance deficits after the deletion of STING in ECs. Subsequently, we subjected the mice to the novel objective recognition (NOR) test, which is an efficient method for investigating memory recognition in mice.^[^ ^26^ ^]^ Compared with wild‐type, *STING^ECKO^* mice showed decreased preference for novel objects (Figure 4F,G), suggesting that their recognition memory was impaired after the deletion of STING in ECs. We used the elevated‐plus maze to measure whether *STING^ECKO^* mice exhibited anxiety‐like behavior. The results showed no significant difference in the open and closed arms (Figure 4H,I), suggesting that *STING^ECKO^* mice showed no anxiety‐like behavior. Previous studies have indicated that oligodendrocyte dysfunction was closely associated with depression.^[^ ^27^ ^]^ Thus, we conducted forced swimming tests to analyze whether *STING^ECKO^* mice exhibited depression‐like behaviors. The results showed no significant difference in the immobility time between *STING^WT^* and *STING^ECKO^* mice (Figure 4J), suggesting that *STING^ECKO^* mice have no depression‐like behaviors. Overall, these results indicate that the deletion of endothelial STING leads to locomotion, and object memory recognition defects in mice.

### 2.6 Deletion of Endothelial STING Disrupts Angiogenesis by Inhibiting Cholesterol Synthesis

**Figure 5:**
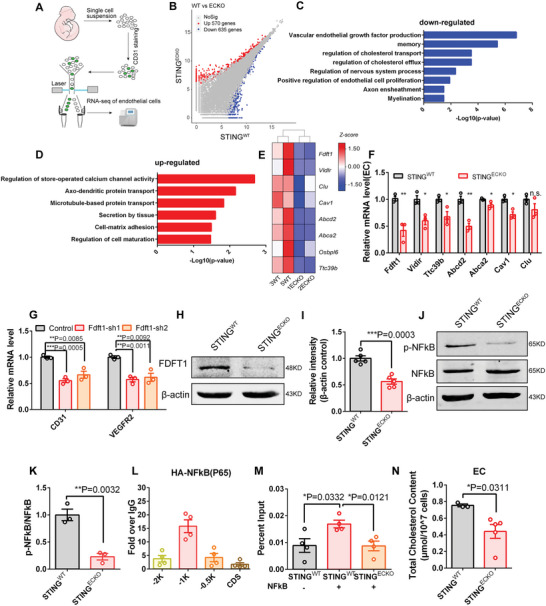
Loss of endothelial STING disrupts angiogenesis through inhibiting cholesterol synthesis. A) Schematic overview of the strategy used to sort ECs in cell suspensions from *STING^WT^* and *STING^ECKO^* mice. B) Volcano diagram shows that the genes whose expression level was significantly changed in ECs from *STING^ECKO^* mice. C,D) Gene Ontology (GO) analysis of biological processes related to the downregulated and upregulated genes after loss of STING in endothelial cells. E) Heat map of 8 differentially expressed genes related to cholesterol synthesis and metabolism from the RNA‐Seq of brain ECs. F) RT‐PCR analysis confirming the availability of downregulated genes at E18 in isolated *STING^ECKO^* mice brain ECs. ^*^ *P* < 0.05, ^* *^ *P* < 0.01 (mean ± SEM, unpaired two‐tailed Student's *t*‐test, n = 3 mice each group). G) RT‐PCR analysis showing the mRNA expression level of CD31 and VEGFR2 was reduced after the knockdown of FDFT1 in primary ECs. ^* *^ *P* < 0.01, ^* * *^ *P* < 0.001 (mean ± SEM, unpaired two‐tailed Student's *t*‐test, n = 3 mice each group). H) Western blot analysis of the expression levels of FDFT1 at E18 in *STING^ECKO^* mice brain primary ECs. β‐actin was detected as a loading control. I) Statistics of relative intensity of FDFT1 showing the decreased expression of FDFT1 in *STING^ECKO^* isolated brain endothelial cells. ^* * *^ *P* < 0.001 (mean ± SEM, unpaired two‐tailed Student's *t*‐test, n = 5 mice each group). J) Western blot analysis of the expression levels of p‐NFκB and NFκB at E18 in *STING^ECKO^* mice brain primary ECs. β‐actin was detected as a loading control. K) Statistics showing the decreased ratio of p‐NF𝜅B and NF𝜅B in *STING^ECKO^* brain endothelial cells. ^* *^ *P* < 0.01 (mean ± SEM, unpaired two‐tailed Student's *t*‐test, n = 3 mice each group). L) ChIP‐qPCR showing NF‐𝜅B binds enrichment on indicated regions of FDFT1 gene. We performed CHIP using a mouse brain endothelial cells Line (bEnd.3). (n = 4 independent chromatin samples). M) ChIP‐qPCR showing the enrichment of NF‐𝜅B on promoters of FDFT1 with or without loss of STING in ECs. We performed CHIP using brain primary endothelial cells. The expression of HA‐ NFκB in primary endothelial cells was obtained by packaging viruses and infecting primary cells. ^*^ *P* < 0.05 (n = 4 independent chromatin samples). N) Statistics showing cholesterol levels were reduced in *STING^ECKO^* mice brain endothelial cells. ^*^ *P* < 0.05 (*STING^WT^* n = 3 mice; *STING^ECKO^* n = 5 mice). Data are represented as means ± SEM. unpaired two‐tailed Student's *t‐*test; At least three biological replicates are shown. ^*^ *P* < 0.05, ^* *^ *P* < 0.01, ^* * *^ *P* < 0.001.

To elucidate the molecular basis of endothelial STING deletion, we conducted RNA sequencing (RNA‐seq) of WT or STING‐deficient ECs in mice brains (**Figure** **5**A). The RNA‐seq analysis showed that STING deletion in brain ECs induced the expression of 570 genes and reduced the expression of 635 genes (Figure 5B). According to Gene Ontology (GO) analysis, the downregulated genes were enriched in terms related to cholesterol transport, cholesterol efflux, and regulation of ECs proliferation, and upregulated genes were enriched in terms related to secretion by tissue and cell‐matrix adhesion (Figure 5C,D). These results implied that STING might play a role in cholesterol synthesis, which plays a key role in regulating angiogenesis.^[^ ^28^ ^]^ We selected the differential genes related to cholesterol synthesis and metabolism from the RNA‐Seq of brain ECs (Figure 5E) and verified the expression levels of obviously downregulated genes by RT‐PCR (Figure 5F). In order to further elucidate the functions of downstream genes, we constructed knockdown vectors targeting FDFT1, ABCD2, and VLDLR, which were significantly downregulated genes in *STING^ECKO^* mice of brain ECs at E18. The results showed that the expression level of CD31 and VEGFR2 have no obvious change after the knock down of ABCD2 and VLDLR in primary brain endothelial cells (Figure S5A,B, Supporting Information), while the knockdown of FDFT1 reduced the expression level of CD31 and VEGFR2 (Figure 5G), suggesting that FDFT1 has a STING KO‐like phenotype. Thus, we chose FDFT1 as a promising candidate molecule. Immunofluorescent staining confirmed that FDFT1 was expressed in ECs (Figure S5C, Supporting Information). Western blotting analysis also further proved that the expression of FDFT1 was obviously reduced in knockout ECs (Figure 5H,I). And we have examined the protein expression and localization of CD31 and VEGFR2 proteins after knockdown of FDFT1. The results showed that knockdown of FDFT1 reduce the expression of CD31 and VEGFR2, suggesting that FDFT1 affected angiogenesis (Figure S5D–I, Supporting Information). We then explored the regulation of FDFT1 expression by STING, and screened several candidates, including NF‐𝜅B, TBK1, IKK𝛽, which can interact with STING and regulate gene expression.^[^ ^29^, ^30^ ^]^ We found that phosphorylated NF‐𝜅B (p65) and phosphorylated IKK𝛽 were significantly downregulated, while the activation of TBK1 was not affected in ECs of *STING^ECKO^* mice (Figure 5J,K; Figure S5J–L, Supporting Information). To validate whether NF‐𝜅B activates FDFT1 expression, we conducted the chromatin immunoprecipitation (ChIP) experiment. The results showed that NF‐𝜅B mainly bound to the FDFT1 promoter 1 kb upstream of the transcriptional start site (Figure 5L). Furthermore, we found that the binding enrichment of NF‐𝜅B on the FDFT1 promoter reduced after the deletion of STING in ECs (Figure 5M), suggesting that STING regulated FDFT1 expression through NF‐𝜅B signaling. Further, we examined whether the induction of FDFT1 caused by STING deficiency is NFκB dependent by adding BAY11‐7085 (NF‐κB inhibitor) and Betulinic acid (NF‐κB activator). Western blot results showed that STING promotes FDFT1 expression through the NFκB signaling pathway (Figure S5M–O, Supporting Information). FDFT1 is a critical enzyme involved in the first stage of cholesterol synthesis, which plays important roles in cell proliferation and development.^[^ ^31^, ^32^ ^]^ Thus, we measured the total cholesterol content of isolated brain ECs, brain, and serum in *STING^WT^* and *STING^ECKO^* mice. Our results showed that the total cholesterol content was reduced in ECs and brain tissue but no obvious difference was observed between the serum of *STING^ECKO^* and *STING^WT^* mice (Figure 5N; Figure S5P,Q, Supporting Information). Knockdown of FDFT1in ECs reduced the total cholesterol content in ECs (Figure S5R, Supporting Information). We found that the overexpression of FDFT1 rescued the impaired proliferation of ECs caused by STING deletion (Figure S6A–C, Supporting Information). We treated STING‐deficient endothelial cells with exogenous cholesterol in vitro, and detected cell proliferation with BrdU and Ki67 immunofluorescence staining. The results showed that exogenous cholesterol treatment in STING‐deficient ECs rescues endothelial cell proliferation (Figure S6D,E, Supporting Information), further indicating that STING facilitates angiogenesis by promoting cholesterol synthesis. Overall, these results suggest that the loss of endothelial STING disrupts angiogenesis by inhibiting cholesterol synthesis.

To substantiate the link between decreased endothelial FDFT1 and impaired oligodendrogenesis in *STING^ECKO^* mice, we overexpressed FDFT1 (adeno‐associated virus, pAAV‐FdFt1 AAV.BI30) by endothelial cell high‐efficiency AAV.BI30 in vivo.^[^ ^33^ ^]^ CD31‐positive ECs showed effective AAV.BI30 vector‐mediated GFP transduction (Figure S6F, Supporting Information). Western blot analysis confirmed that the AAV‐FDFT1 induced overexpression of FDFT1 in isolated primary ECs. In order to verify whether FDFT1 overexpression affects the expression of IL17D in endothelial cells, we detected the expression level of IL1D after overexpression of AAV‐FDFT1 by western blotting, and found that overexpression of FDFT1 inhibited IL17D expression in endothelial cells (Figure S6G–I, Supporting Information). We next evaluated whether restored FDFT1 could improve impaired angiogenesis in *STING^ECKO^* mice. The results showed that AAV‐FDFT1 injection increased vessel length in *STING^ECKO^* mice (Figure S6J,K, Supporting Information). AAV‐FDFT1 injection also up‐regulated myelin MBP expression in STING*^ECKO^* mice (Figure S6L,M, Supporting Information). We also found that AAV‐FDFT1 injection up‐regulated the percentage of CC1^+^ cells among OLIG2 cells in the corpus callosum of *STING^ECKO^* mice (Figure S6N,O, Supporting Information). Taken together, these evidences FDFT1 in ECs regulates angiogenesis and oligodendrogenesis in vivo.

### 2.7 Deletion of Endothelial STING Inhibits Oligodendrogenesis by Increasing IL17D Signaling

**Figure 6:**
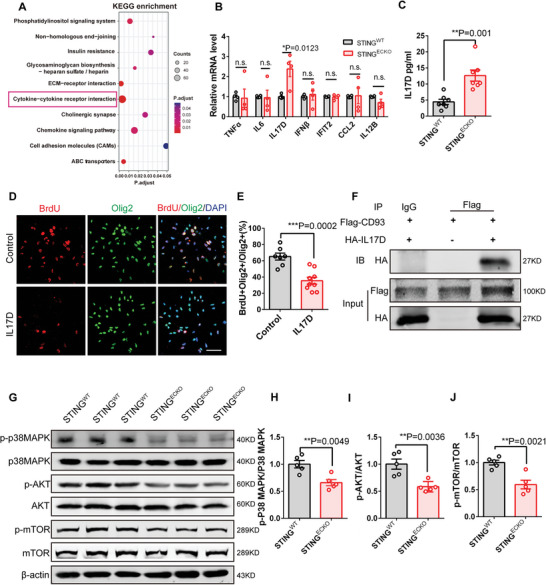
Deletion of endothelial STING inhibits oligodendrogenesis by increasing IL17D signaling. A) KEGG pathway analysis on the RNA‐seq dataset shows potential responses involving cytokine‐cytokine receptor interaction. B) RT‐PCR analysis showing IL17D was significantly upregulated at E18 in isolated *STING^ECKO^* mice brain ECs. n.s., not significant, ^*^ *P* < 0.05 (mean ± SEM, unpaired two‐tailed Student's *t*‐test, n = 4 mice each group). C) ELISA analysis by collecting supernatant in isolated *STING^WT^* and *STING^ECKO^* mice brain ECs showing increased IL17D in endothelial STING knockout mice. ^* *^ *P* < 0.01 (mean ± SEM, unpaired two‐tailed Student's *t*‐test, n = 7 Imice each group). D) Confocal immunofluorescence image of BrdU^+^ Olig2^+^ in oligodendrocyte precursor cells. Scale bar, 50 µm. E) Quantification showing IL17D inhibited the proliferation of oligodendrocyte progenitor cells. ^* * *^ *P* < 0.001 (mean ± SEM, unpaired two‐tailed Student's *t*‐test, Control: n = 7, IL17D: n = 9 from 3 independent experiments). F) Immunoprecipitation (IP) experiment showing the interaction between IL17D and CD93. G) Western blot analysis of the expression levels of p‐P38MAPK, P38MAPK, AKT, p‐AKT, mTOR, and p‐mTOR at P7 in *STING^WT^* and *STING^ECKO^* mice corpus callosum. β‐actin was detected as a loading control. H) Statistics showing the decreased ratio of p‐P38MAPK and P38MAPK in *STING^ECKO^* mice corpus callosum. ^* *^ *P* < 0.01 (mean ± SEM, unpaired two‐tailed Student's *t*‐test, n = 5 mice each group). I) Statistics showing the decreased ratio of p‐AKT and AKT in *STING^ECKO^* mice corpus callosum. ^* *^ *P* < 0.01 (mean ± SEM, unpaired two‐tailed Student's *t*‐test, n = 5 mice each group). J) Statistics showing the decreased ratio of p‐mTOR and mTOR in *STING^ECKO^* mice corpus callosum. ^* *^ *P* < 0.01 (mean ± SEM, unpaired two‐tailed Student's *t*‐test, n = 5 mice each group). Data are represented as means ± SEM. unpaired two‐tailed Student's *t‐*test; At least three biological replicates are shown. n.s., not significant, ^*^ *P* < 0.05, ^* *^ *P* < 0.01, ^* * *^ *P* < 0.001.

The loss of endothelial STING affects both angiogenesis and oligodendrogenesis. To explore the molecular signaling mechanisms of ECs controlling environmental signals for the response of OPCs, we conducted Kyoto Encyclopedia of Genes and Genomes (KEGG) pathway analysis on the RNA‐seq dataset and discovered potential responses involving cytokine‐cytokine receptor interaction (**Figure** **6**A). Previous studies have demonstrated that arterial endothelial cells respond to modified lipoproteins by getting activated and producing chemokines or cytokines in atherosclerosis,^[^ ^34^, ^35^ ^]^ implying a correlation between cholesterol and cytokine. Previous studies have shown that cytokine secreted by other cells, such as migrating neurons and B‐1a lymphocytes, regulate the formation of oligodendrocytes in the developing mammalian brain.^[^ ^36^, ^37^ ^]^ So, we further examined whether vascular endothelial cell cytokines regulate oligodendrogenesis. We screened innate immune‐related cytokines and found that IL17D was most significantly upregulated in the STING‐deficient ECs compared with *STING^WT^* cells (Figure 6B). Western blotting analysis also further indicated that the expression levels of IL17D increased after the deletion of STING in ECs (Figure S7A,B, Supporting Information). Immunofluorescence staining confirmed the decrease in intracellular cholesterol and the increase in IL17D in the STING‐deficient ECs (Figure S7C,D, Supporting Information). Furthermore, the IL17D content increased in conditioned media from isolated ECs in *STING^ECKO^* mice (Figure 6C). We found that exogenous cholesterol treatment of ECs reduced the expression level of IL17D (Figure S7E, Supporting Information), suggesting that the excessive production of IL17D was attributed to cholesterol dysregulation in ECs. We found that exogenous IL17D treatment reduced the expression of OLIG2 (Figure S7F,G, Supporting Information) and inhibited the proliferation of OPCs (Figure 6D,E). Previous research has shown that CD93 is a cell‐surface type I transmembrane protein with an amino‐terminal C‐type lectin‐like domain, which has been identified as a receptor for IL‐17D.^[^ ^38^ ^]^ Thus, we first examined the expression levels of CD93 in OPCs, neurons, astrocytes, and ECs and found that CD93 expression in OPCs was the highest (Figure S7H, Supporting Information). To validate whether IL17D interacted with CD93 to regulate the response of OPCs, we conducted co‐immunoprecipitation (Co‐IP) assays. The results showed that FLAG‐tagged CD93 clearly pulled down HA‐tagged IL17D, suggesting a direct interaction between IL17D and CD93 (Figure 6F). Considering that the P38MAPK and p‐AKT/mTOR signaling pathway is a classical signaling pathway that regulates the expression of genes related cell growth and cell differentiation.^[^ ^39^, ^40^ ^]^ So, we explored the influence of IL17D signaling on oligodendrocytes and the effects on downstream p‐P38MAPK, p‐AKT and p‐mTOR in OPCs. We found that the expression levels of p‐P38MAPK, p‐AKT, and p‐mTOR were reduced in the corpus callosum of *STING^ECKO^* mice (Figure 6G–J). To demonstrate the direct involvement of CD93 in transmitting the IL17D signal, we conducted experiments in OPCs (oligodendrocyte progenitor cells) by blocking the function of CD93. When the function of CD93 was blocked in OPCs, we observed a rescue of the phenotype characterized by reduced expression levels of p‐P38MAPK, p‐AKT, and p‐mTOR (Figure S7I,J, Supporting Information). The results suggested that IL17D transmits signals to OPCs through the CD93 receptor.

### 2.8 Overexpression of Endothelial FDFT1 or AKT Activator Rescues Impaired Oligodendrogenesis

**Figure 7:**
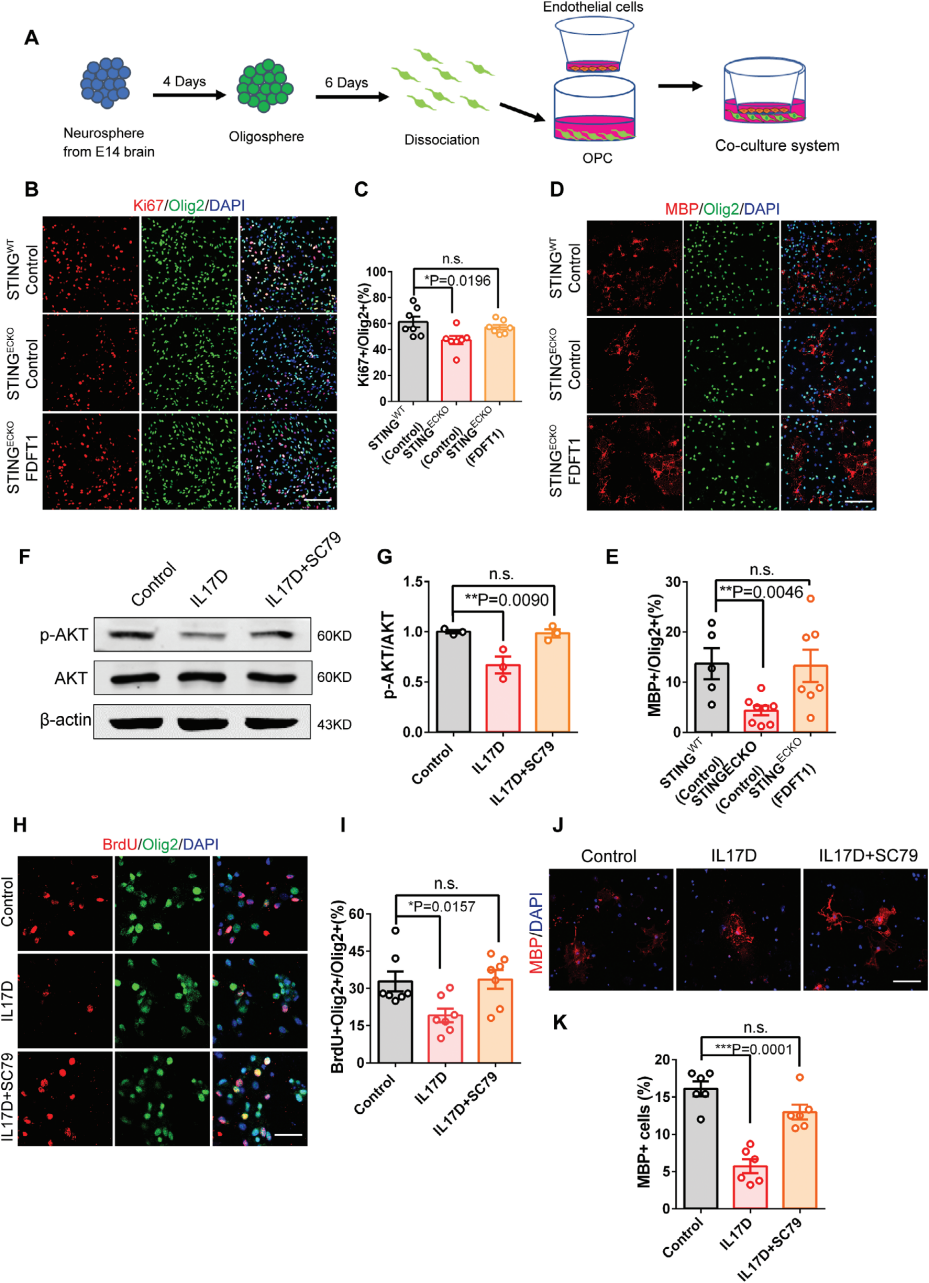
Endothelial FDFT1 Overexpression or AKT activator rescue impaired oligodendrogenesis. A) The diagram showing endothelial cells were co‐cultured with OPCs in transwell membranes. B) Confocal immunofluorescence image showed that endothelial FDFT1 can rescue the proliferation of damaged oligodendrocyte progenitor cells caused by endothelial STING deletion. Scale bar, 100 µm. C) Quantification of the percent of Ki67^+^ Olig2^+^cells. ^*^ *P* < 0.05 (mean ± SEM, one‐way ANOVA with Dunnett's multiple‐comparison correction, n = 7 each group from 3 independent experiments). D) Confocal immunofluorescence image showed that endothelial FDFT1 can rescue differentiation of damaged oligodendrocyte progenitor cells caused by endothelial STING deletion. Scale bar, 100 µm. E) Quantification of the percent of MBP^+^ Olig2^+^cells. ^* *^ *P* < 0.01 (mean ± SEM, one‐way ANOVA with Dunnett's multiple‐comparison correction, *STING^WT^* (Control): n = 5, *STING^ECKO^* (Control): n = 8 sample, *STING^ECKO^* (FDFT1): n = 7 sample from 3 independent experiments). F) Western blot analysis of the expression levels of p‐AKT and AKT after adding AKT activator SC79 two days. β‐actin was detected as a loading control. G) Statistics showing the decreased ratio of p‐AKT and AKT after adding AKT activator SC79 two days. ^* *^ *P* < 0.01 (mean ± SEM, unpaired two‐tailed Student's *t*‐test, n = 3 each group). H) Confocal immunofluorescence image showed that AKT activator SC79 can rescue the proliferation of damaged oligodendrocyte progenitor cells. Scale bar, 20 µm. I) Quantification of the percent of BrdU^+^ Olig2^+^cells. ^*^ *P* < 0.05 (mean ± SEM, one‐way ANOVA with Dunnett's multiple‐comparison correction, n = 7 sample each group). J) Confocal immunofluorescence image showed that AKT activator SC79 can rescue myelination. Scale bar, 50 µm. K) Quantification of the percent of MBP^+^ cells. ^* * *^ *P* < 0.001 (mean ± SEM, one‐way ANOVA with Dunnett's multiple‐comparison correction, n = 6 sample each group). Data are represented as means ± SEM. unpaired two‐tailed Student's *t‐*test and one‐way ANOVA with Dunnett's multiple‐comparison correction. At least three biological replicates are shown. n.s., not significant, ^*^ *P* < 0.05, ^* *^ *P* < 0.01, ^* * *^ *P* < 0.001.

To determine whether the overexpression of endothelial FDFT1 could rescue the impaired oligodendrogenesis, we prepared primary brain ECs and co‐cultured them with OPCs prepared by the dissociation of oligospheres in transwell membranes (**Figure** **7**A). We found that the overexpression of FDFT1 rescued the impaired proliferation and differentiation of OPCs caused by endothelial STING deletion (Figure 7B–E). As the treatment of OPCs with exogenous IL17D affected the expression of p‐AKT, we further tested whether reductive Akt signaling might be responsible for the impaired OPCs response. Subsequently, we selected cell‐permeable AKT activator SC79 to demonstrate the function of AKT in OPCs. Western blotting confirmed the efficiency of SC79 in AKT activation (Figure 7F,G). After 2 days of treatment with exogenous IL17D and SC79, we conducted immunofluorescence staining. The results showed that AKT activation promotes the proliferation of OPCs under the exposure to IL‐17D (Figure 7H,I). In order to detect whether SC79 rescued myelination, we performed MBP immunofluorescent staining after 2 days of treatment with exogenous IL17D and SC79. The results showed that AKT activation promotes myelination under the exposure to IL‐17D (Figure 7J,K). In summary, our study provided insights into the regulatory link between ECs and OPCs response. The loss of endothelial STING disrupted angiogenesis by altering cholesterol synthesis, resulting in an increase in IL17D, which interacted with the CD93 receptor by regulating the P38MAPK and AKT/mTOR signal pathway to transmit EC signals into OPCs during brain development (Figure S8, Supporting Information).

## 3 Discussion

The coordination of oligovascular coupling is important for maintaining tissue stability during brain development.^[^ ^41^ ^]^ In this study, we proposed a novel biological mechanism describing the significant role of brain endothelial STING in driving the OPCs response during brain development. Deletion of endothelial STING signaling affects angiogenesis by inhibiting cholesterol synthesis and disrupts oligodendrogenesis and myelination. Overall, our results indicated that the brain vasculature is an important niche for OPCs during cortical development.

STING signaling has emerged as a key mediator of inflammation during infection, cellular stress, and tissue damage.^[^ ^42^ ^]^ Unlike the well‐characterized roles of STING, we confirmed a central role of endothelial STING in the regulation of metabolic homeostasis during brain development. STING deficiency in ECs disrupts angiogenesis by inhibiting cholesterol synthesis. Recent studies have demonstrated an association between STING and metabolic dysfunction. Obesity results in STING activation and production of cytokines, which leads to insulin tolerance and metabolic disorders.^[^ ^43^ ^]^ STING regulates polyunsaturated fatty acid (PUFA) metabolism by inhibiting the rate‐limiting enzyme activity of fatty acid desaturase 2 (FADS2).^[^ ^11^ ^]^ Furthermore, STING also acts as a proliferative inhibitor in T lymphocytes of the adaptive immune system.^[^ ^44^ ^]^ Therefore, increasing evidence has shown that STING has other functions in addition to immunity.

Blood vessels are lined by a monolayer of ECs, which provide dynamic and selective barriers between blood and the surrounding tissues.^[^ ^45^ ^]^ Increasing evidence has indicated that energy metabolism in ECs plays an important role in regulating angiogenesis. The activation of cGAS‐STING signaling is usually accompanied by the activation of NF‐κB. However, the specific mechanism by which STING activates NF‐κB signaling is still controversial. Recent study confirms the essential involvement of NF‐kB activation in certain biological functions mediated by STING, which are less impaired when IFN induction is selectively dampened.^[^ ^46^ ^]^ STING may activate NF‐kB in the framework of supramolecular organizing centers (SMOCs), which location‐specific higher‐order signaling complexes in which increased local concentrations of signaling components promote the intrinsically weak allosteric interactions necessary for enzyme activation.^[^ ^47^ ^]^ We found that phosphorylated NF‐𝜅B (p65) and phosphorylated IKK𝛽 were significantly downregulated, while the activation of TBK1 was not affected in ECs of STING*^ECKO^* mice, suggesting that STING may activate NF‐κB independently of TBK1. We have confirmed that STING promotes FDFT1 expression through the NFκB signaling pathway. It is noteworthy that FDFT1 is an important enzyme involved in cholesterol synthesis. The *Tie2* gene is expressed vascular endothelial cells and is also present on circulating hematopoietic cells, including megakaryocytes and neutrophils.^[^ ^48^ ^]^ Therefore, we cannot rule out whether hematopoietic stem cell‐derived cells influence OPCs proliferation and differentiation. With this question in mind, we further examined the effect of FDFT1 with endothelial cell high‐efficiency AAV.BI30. In our study, we have observed a decrease in cholesterol levels within endothelial cells, which may be attributed to the reduction of FDFT1. The specific mechanisms underlying this phenomenon still require further investigation.

Brain cholesterol is locally synthesized, with varying synthesis capacities across different cell types. oligodendrocyte precursor populations in spinal cord exhibit higher levels of cell‐autonomous cholesterol biosynthesis than the equivalent stage precursors in the brain.^[^ ^49^ ^]^ The proliferation of oligodendrocyte precursor cells in the brain relies on the uptake of cholesterol from neurons.^[^ ^49^, ^50^ ^]^ Oligodendrocytes synthesize cholesterol at a high rate during myelination after birth. Cholesterol in endothelial cells plays an important role in maintaining endothelial cell function and angiogenesis.^[^ ^28^, ^51^ ^]^ So, cholesterol has an important physiological function in the brain. However, it is still unknown whether cholesterol or its metabolites in endothelial cells can travel beyond blood vessels and affect the function of other cells in the brain.

Furthermore, our study demonstrated that the perturbation of metabolic homeostasis in ECs increases the expression levels of cytokine IL17D, and exogenous cholesterol treatment of ECs reduces the expression level of IL17D. These results suggest that the inhibition of one pathway is followed by the perception of the other pathway. However, the regulation mechanism of the increase in IL17D still needs to be validated in future studies. The activation of NFκB does not affect the expression of IL‐6 and TNF‐α in endothelial cells after loss of STING in ECs. One possible reason is the involvement of other transcription factors and signaling pathways may influence the expression of IL‐6 and TNF‐α, such as AP‐1, STAT, and CREB.^[^ ^52^, ^53^, ^54^, ^55^ ^]^ Previous studies have shown that the blood‐vascular system of the brain regulated the fate determination of neural precursor cells and migration of OPCs.^[^ ^5^, ^56^ ^]^ We observed that the loss of endothelial STING disrupts angiogenesis, leading to disruption of cytokine release in the CNS. our data demonstrated that EC‐derived IL17D was bound on the CD93 receptor of OPCs to regulate P38MAPK and AKT/mTOR signaling, which controls oligodendrogenesis. P38MAPK is expressed in the oligodendrocyte lineage, which regulates the development of oligodendrocyte.^[^ ^57^ ^]^ These results are consistent with a previous study stating that Akt signaling drives the differentiation and myelination of oligodendrocyte through the mTOR pathway.^[^ ^58^ ^]^ IL‐17D is the least understood member of the IL‐17 family, which interacts with CD93 in regulating intestinal homeostasis.^[^ ^38^ ^]^ Our results indicated that the EC‐derived IL17D cytokines regulate OPCs response during brain development. In conclusion, our results reveal that the endothelial‐derived STING signal regulates both angiogenesis and the proliferation and differentiation of OPCs. Our study provides new insights into the regulatory link between blood vessels and OPCs.

## 4 Experimental Section

### Animals

Pregnant ICR and C57BL/6 mice were obtained from Vital River Laboratories. The STING*^flox/flox^* mice^[^ ^59^ ^]^ and the TEK‐Cre (Tie2‐Cre)(NR‐KI‐210133) mice were obtained from the Shanghai Model Organisms Center. Using homologous recombination principles and ES cell targeting, we performed flox modification on the STING gene. STING floxed mice were crossed with C57BL/6J mice, and then the F1 offsprings were bred to obtain *STING^flox/flox^* mice. *STING^ECKO^* mice were generated by crossing *STING^flox/flox^* mice with Tie2‐Cre mice. Mice were bred and maintained at 22–25 °C with a 12 h light/dark cycle, and provided adequate food and water. All animal experiments were conducted in accordance with the guidelines of the Animal Care and Use Committee of the Institute of Zoology, Chinese Academy of Sciences (IOZ20190035).

### Plasmid Constructs

STING cDNA was acquired by PCR and subcloned into the 3×Flag‐tagged PCDH (CD511B‐1; System Biosciences) vector. cGAS shRNA were cloned into the pSicoR‐GFP vector. The sequences were as follows: GAGATTGAAACGCAAAGATAT. FDFT1 cDNA was acquired by PCR and subcloned into the 3xHA‐tagged PCDH vector. FDFT1 shRNA were cloned into the pSicoR‐GFP vector. The sequences were as follows: FDFT1‐shRNA1: GTGTTTAACTTCTGTGCTATT; FDFT1‐shRNA2: CAGTGCTTGAATGAACTCATA.

### Cell Culture

Human embryonic kidney 293T cells (HEK293FT) were cultured in DMEM (Gibco,11995‐065) supplemented with 10% FBS (Gibco,16000044), 1% penicillin/streptomycin (Invitrogen, 15070063). The mouse brain endothelial cells(bEnd.3) were cultured in DMEM (Gibco, 11995‐065) containing 10% FBS and 1% penicillin/streptomycin (Invitrogen, 15070063). The medium was changed every three days.

### Isolation and Culture of Primary Mouse Brain Endothelial Cells

Primary mouse brain ECs were isolated and cultured as previously described.^[^ ^60^ ^]^ Briefly, E18 fetal brains or P0 brains were dissected under stereomicroscopy to remove the brainstem, cerebellum, olfactory bulb and the pial membranes, and obtained the cerebral cortex. The brain cortex was digested into cell suspension using 20 U mg^−1^ papain for 5 min at 37 °C. The cells were filtered through a sterile 70‐µm nylon mesh, and then lysed with 2 ml red blood cell lysing buffer (Sigma–Aldrich) for 3 min at room temperature. Finally, cells were cultured on collagen type I (Sigma–Aldrich) coated 6‐Well or 24‐Well plates (costar) in EGM‐2 media (EGM‐2 BulletKit; Lonza, CC‐3162). The medium was replaced with fresh medium every 3 days.

### Isolation and Culture of Primary Mouse Oligodendrocyte Precursor Cells

Primary mouse oligodendrocyte precursor cells were isolated and culture as previously described.^[^ ^61^ ^]^ E15 fetal brains were removed to remove the brainstem, cerebellum, olfactory bulb and the pial membranes, and removed the medial portion and retained the lateral part of the cerebral cortex. The brain cortex was digested into cell suspension using 20 U mg^−1^ papain for 5 min at 37 °C. Cell suspensions were filtered through a 70 µm strainer and seeded 24‐Well plates (costar) in neurosphere growth medium composed of DMEM/F12(Gibco, 11330‐032) supplemented with 20 ng ml^−1^ bFGF and 20 ng ml^−1^ EGF. The medium was replaced half of the medium with fresh neurosphere growth medium every 2 days for 4 consecutive days. And then, the medium was replaced with one‐fourth of the former medium with fresh oligosphere medium composed of DMEM/F12(Gibco, 11330‐032) supplemented with5%FBS (Gibco,16000044), 20 ng ml^−1^ bFGF and 20 ng ml^−1^ PDFG‐AA every 2 days for 6 consecutive days. Oligospheres were digested into cell suspension using with 0.5 ml of 0.05% trypsin at 37 °C for 5 min. The cells were cultured in poly‐l‐lysine–precoated (Sigma‐Aldrich, P3655; 0.1 mg ml^−1^ in PBS) coated 6‐Well or 24‐Well plates (costar) in proliferation medium composed of DMEMF/12 medium (Gibco, 11330‐032),

50×B27 supplement without VA (Invitrogen, 12587010), 100×N2 supplement (Gibco 17502‐048), 100×Gluta MAX (Invitrogen, 35050061), 100×sodium pyruvate (Gibco, 11360070), 10 nM hydrocortisone (CC‐4031L), 5 µg ml^−1^ insulin (CC‐4021L), 10 ng ml^−1^ EGF (Invitrogen, PHG0311), 10 ng ml^−1^ bFGF (Invitrogen, PHG0026), 10 ng ml^−1^ PDFG‐AA (PeproTech,100‐13A) and 100×penicillin/streptomycin (Invitrogen). Differentiation of OPC into mature OLs was induced by adding CNTF (PeproTech, 450‐13) and T3 (Sigma–Aldrich, T6397) into the culture medium, with the medium changed every 3 days.

For the co‐culture experiments, brain endothelial cells were cultured on collagen type I (Sigma‐Aldrich) coated 6‐Well in EGM‐2 media (EGM‐2 Bullet Kit; Lonza, CC‐3162). When the endothelial cells reached ≈80% confluence, endothelial cells were infected with FDFT1 lentivirus. After 3 days of viral infection, we co‐cultured the endothelial cells with pre‐prepared oligodendrocyte progenitor cells. The co‐culture was maintained under proliferative conditions for 3 days, or differentiation conditions for 4 days before proceeding with subsequent experiments.

### Isolation and Culture of Primary Astrocytes and Neurons

Primary astrocytes and neurons were isolated and cultured as previously described.^[^ ^62^ ^]^ In brief, the fetal brain was dissected under stereomicroscopy to remove the brainstem, cerebellum, and olfactory bulb and obtain the cerebral cortex. The brain cortex was digested into cell suspension using 20 U mg^−1^ papain (Worthington, Ls003119) for 5 min at 37 °C. Then, cells were purified by washing three times with DMEM (Gibco, 11995‐065). After the supernatant was removed, fresh culture medium was added to prepare cell suspension. Cell suspensions were filtered through a 70 µm strainer. Neuronal culture medium composed of Neurobasal medium, 1% heat‐inactivated horse serum (HS), 0.5 mM glutamine, 1% penicillin–streptomycin (P/S), 0.04% sodium bicarbonate, 33 mM glucose, and 2% B27 supplement. Glial culture medium composed of DMEM supplemented with 10% FBS and 1% P/S.

### BrdU Labeling

For BrdU labeling, pregnant mice were injected with 50 mg kg^−1^ BrdU (sigma) 2 h before embryonic brains were extracted and fixed at E18. For cells BrdU labeling, cells were treated with 10µ g ml^−1^ BrdU2 h before immunofluorescence cell staining.

### TUNEL Assays

Apoptosis cells were detected by TUNEL assay using a TransDetectIn Situ Fluorescein TUNEL Cell Apoptosis Detection Kit (Transgen Biotech, China) according to the protocol. Briefly, Brain slices fixed in 4% paraformaldehyde (PFA) for 30 min at room temperature, and washed three times with PBS and then treated with 0.1% Triton X‐100 in PBS for 5 min at room temperature. The sections were incubated with the TUNEL reaction buffer, containing 50 µl of the labeling solution and 2 µl of the enzyme solution at 37 °C for 1 h in a humidified and dark atmosphere. After washing three times, the specimens were observed with a fluorescence microscope with an excitation wavelength in the range of 450–500 nm.

### AAV Vector Administration in Mice

AAV.BI30 is a high‐efficiency AAV for endothelial cell transduction throughout the central nervous system.^[^ ^33^ ^]^ The AAV.BI30 vectors or pAAV‐FDFT1‐AAV.BI30 was constructed by Vigene Bioscience (Shandong, China) and injected intravenously via the retro‐orbital route to P2 neonatal mice at a dose of 5–6 ×10^12^ vg per mouse. The retro‐orbital injections were performed as described previously.^[^ ^63^, ^64^ ^]^ These AAV‐injected mice were performed immunofluorescent staining four weeks after AAV injection.

### Immunostaining

Brain slices and cultured cell immunostaining was performed as previously described.^[^ ^65^ ^]^ Briefly, brain slices fixed in 4% paraformaldehyde (PFA) for 30 min at room temperature, and washed three times with phosphate buffered saline (PBS) containing 1% Triton X‐100 (1% PBST). Brain slices were blocked with 5% bovine serum albumin (in 1% PBST) for 1 h, and then incubated with primary antibodies at 4 °C overnight. For cells staining, cultured cells were washed with PBS for 5 min and fixed in 4% PFA for 20 min at room temperature, and washed three times with phosphate‐buffered saline (PBS) containing 0.1% Triton X‐100 (0.1% PBST). The cultured cells were blocked with 5% BSA (in 0.1% PBST) for 1 h, and then incubated with primary antibodies at 4 °C overnight. The following day, the samples to be visualized were incubated with secondary antibodies for 1.5 h at room temperature. Confocal images were captured by a Carl ZeissLSM880 confocal microscope.

### Western Blotting and Co‐Immunoprecipitation

Western blotting procedures were performed as described previously.^[^ ^66^ ^]^ Briefly, the protein was extracted from brain tissues or cells with RIPA lysis buffer (Solarbio) containing a 1% protease inhibitor cocktail and phenylmethylsulfonyl fluoride (PMSF). Subsequently, protein concentrations were determined using a Pierce BCA Protein Assay Reagent (Thermo Scientific). Next, Protein samples were separated by SDS–PAGE gel and transferred onto polyvinylidene fluoride (PVDF) membranes. The membranes were blocked with 5% milk with PBST (0.05% Tween 20 in PBS) for 1 h at room temperature and incubated with primary antibodies at 4 °C overnight. The following day, the bands were visualized and analyzed by the Image Studio Ver 5.2 software after secondary antibodies for 1.5 h at room temperature.

For co‐immunoprecipitation, protein samples from transfected cells were lysed in CO‐IP lysis buffer (Beyotime Biotechnology) containing 1% protease inhibitor cocktail and PMSF. The supernatant was incubated with anti‐HA‐tag or anti‐Flag‐tag magnetic beads (MBL) at 4 °C overnight. After washing three times with cold wash buffer, the bound proteins were analyzed by western blot.

### Chromatin Immunoprecipitation

Cultured cells were maintained with 1% fresh formaldehyde solution for 10 min at room temperature for generating the cross‐link. Then, 2.5 m glycine solution was added to each well to terminate the cross‐linking reaction. After washing twice with ice‐cold PBS, cells were resuspended in lysis buffer 1 (50 mm HEPES–KOH, pH 7.5, 140 mm NaCl, 1 mm EDTA, 0.5% NP‐40, 0.25% Triton, 10% glycerol,1×PMSF, and 1 × cocktail) for 10 min at 4 °C. Next, cells were washed with lysis buffer 2 (10 mm Tris–HCl, pH 8.0, 200 mm NaCl, 0.5 mm EGTA, 1 mm EDTA,1× PMSF, and 1× cocktail) for 10 min at 4 °C. Samples were sonicated in lysis buffer 3(10 mm Tris–HCl, pH 8.0, 1 mm EDTA,100 mm NaCl, 0.5 mm EGTA, 0.1% sodium deoxycholate, 1×PMSF, and 1× cocktail). The lysates were incubated with anti‐HA‐tag magnetic beads (Invitrogen) and anti‐IgG magnetic beads (Invitrogen) overnight at 4 °C. Beads were washed three times each with low‐salt buffer and high‐salt buffer at room temperature and then incubated overnight at 65 °C. After extraction by DNA Gel Extraction Kit (Tiangen), Genomic DNA was performed by RT–PCR. The primers used for real‐time PCR in Table S1 (Supporting Information).

### Antibodies

The following primary antibodies and dilutions were used for immunostaining and western blotting: anti‐STING (Cell Signaling Technology, 13647, Rabbit,1:1000); anti‐BrdU(Abcam, AB6326, Rat, 1:1000); anti‐Ki67 (Abcam, ab15580,Rabbit 1:1000); anti‐biotinylated Isolectin B4 (Vector Laboratories, B‐1205, 1:600); anti‐GFAP (Dako, Z0334, Rabbit,1:3000); anti‐GFAP (Sigma, G6171, Mouse,1:1000); anti‐TUJ1(Sigma, T2200, Rabbit,1:1000); anti‐β‐Actin (Protein tech, 20536‐1‐AP, Rabbit, 1:10000); anti‐β‐Actin (Protein tech; 60008‐1‐Ig Mouse,1:2000); anti‐ALDH1L1 (Abcam, ab56777, Rabbit, 1:2000); anti‐Flag (Sigma, F1804, Mouse, 1:3000), anti‐HA (Cell Signaling Technology, 3724s, Rabbit,1:1000); anti‐ FDFT1(Protein tech, 13128‐1‐AP, Rabbit, 1:1000); anti‐IL17D (biorbyt, orb5539, Rabbit,1:1000); anti‐Olig2(Abcam, ab109186,Rabbit,1:2000); anti‐Olig2(R&D Systems, AF2418,Goat,1:1000); anti‐PDGFRα(Cell Signaling Technology, 3174T, Rabbit,1:1000); anti‐CC1(Millipore, OP80, Mouse,1:1000); anti‐PLP1 (Abcam, ab28486, Rabbit, 1:1000); anti‐MBP (Millipore, MBP386, Rat, 1:1000); anti‐pNF‐κB p65 (Cell Signaling Technology,3033T,Rabbit,1:1000); anti‐NF‐κB p65(Cell Signaling Technology,6956S,Rabbit,1:1000);anti‐p‐P38 MAPK(Cell Signaling Technology, 4511P,Rabbit,1:1000); anti‐P38 MAPK(Cell Signaling Technology, 8690S,Rabbit,1:1000); anti‐AKT(Cell Signaling Technology, 4685S,Rabbit,1:1000); anti‐p‐AKT(Cell Signaling Technology, 3787S,Rabbit,1:1000); anti‐mTOR (Cell Signaling Technology, 2983T, Rabbit, 1:1000); anti‐p‐mTOR (Cell Signaling Technology, 5536S, Rabbit, 1:1000);anti‐IBA1(Wako,019‐19741, Rabbit,1:1000); anti‐Collagen IV(Abcam; ab6586; Rabbit,1:1000); anti‐ZO‐1(Invitrogen, 40–2200,Rabbit,1:500); anti‐Claudin 5 (Invitrogen,35‐2500, Mouse,1:500).

The following secondary florescence antibodies and dilutions for immunostaining: Alexa Fluor 488, Cy3, or Cy5 (Jackson ImmunoResearch,1:1000). DAPI (2 mg ml^−1^; Sigma; D9542) was used for nuclear counterstaining. The following secondary antibodies and dilutions were used for western blotting: Donkey anti‐IgG (LI‐COR Biosciences680LT, Mouse,1:1000); Donkey anti‐IgG (LI‐COR Biosciences680LT, Rabbit,1:1000); Donkey anti‐IgG (LI‐COR Biosciences800LT, Rat,1:1000); Donkey anti‐IgG (LI‐COR Biosciences680LT, Mouse,1:1000); Donkey Anti‐IgG (LI‐COR Biosciences800CW, Rabbit,1:1000); Donkey Anti‐IgG (LI‐COR Biosciences800CW, Mouse,1:1000).

### Electron Microscopy Analysis

Electron microscopy procedures were performed as described previously.^[^ ^67^ ^]^ Briefly, *STING^fl/fl^* and *STING^ECKO^* mice were anesthetized and perfused with cacodylate buffer followed by 2% glutaraldehyde/2% paraformaldehyde. After perfusion, corpus callosum was postfixed for 12 to 24 h at 4 °C in the fixative mixture. Tissues were then first immersed in 1% OsO4 and 1.5%potassium ferricyanide aqueous solution at 4 °C for 2 h. After obtaining, the ultrathin sections were stained by uranyl acetate and lead citrate, and examined by a transmission electron microscope.

### Total Cholesterol (TC) Content Assay

Total cholesterol was extracted from brain endothelial cells, supernatant, serum, and brain tissue by using isopropyl alcohol. Follow manufacturer's experimental procedures, Total cholesterol was quantitatively analyzed by Total Cholesterol (TC) Content Assay Kit (boxbio, AKFA002M).

### ELISA Assay

Brain endothelial cells were isolated from *STING^fl/fl^* and *STING^ECKO^* brain cortices and cultured in endothelial cells medium for 5 days at 37 °C. Follow manufacturer's experimental procedures, IL17D concentrations were quantitatively analyzed by Mouse Interleukin 17D (IL17D) ELISA kit (Feiyue Biotechnology, FY‐EM13693).

### Real‐Time PCR Analysis

The total RNA was extracted from Endothelial STING conditional knockout mouse cerebral cortex, or primary brain endothelial cells by using the Fast Quant RT Kit (Tian gen). The results of real‐time time‐PCR were analyzed by using a SYBR qPCR master mix (Tian gen) and the ABI7500 real‐time PCR system (Applied Biosystems). All primer sequences are listed in Table S2 (Supporting Information).

### RNA‐Sequencing Analysis

Total RNA was extracted from E18 brain endothelial cells of *STING^f/f^* and *STING^ECKO^*. Agilent 2100 Bioanalyze was used to quality controlled and quantified. Then, total RNA was converted to cDNA and bound the library, and RNA‐sequencing analysis was used by the Illumina HiSeq 2500 platform in Annoroad Genomics.

### Behavioral Experiments

All the mice used for behavioral experiments were 8–10 weeks and housed in groups with the mixed genotypes.

### Open‐Field Test

The open‐field was used to analyze the locomotor activity. The open‐field apparatus was a (40 cm x 40 cm x 40 cm) box. The open‐field test was performed by the previously published method.^[^ ^68^ ^]^ Mice were allowed to explore freely for 5 min. The total distance, movement locus, time spent in the center and in the border were recorded and analyzed by the EthoVision XT 14 software.

### Elevated‐Plus Maze

Elevated‐plus maze was conducted as previously described with some modification.^[^ ^69^ ^]^ The apparatus was consisted of two open arms and two closed arms in addition to center zone. The elevated‐plus maze was elevated to a height of 40 cm above the floor. Mice were placed into the center and allowed freely to move for 10 min. Time spent in the open arms and in the closed arms were recorded by the EthoVision XT 14 software.

### Novel Object Recognition Test

The novel object recognition task was used to evaluate the declarative memory, which was independent of the hippocampus.^[^ ^70^ ^]^ The procedure was performed as previously described with modification.^[^ ^71^, ^72^ ^]^ Briefly, the apparatus was a 40cm x 40 x cm x 40 cm box. Mice were placed an empty area to habituate for 10 min. After 2 h, two identical objects (orange‐colored squares) were placed in the testing box. Mice were placed into the box to freely explore for 10 min. After 24 h, one object was alternatively replaced with a novel object, a cream‐colored cylinder. Mice were returned into the box to freely explore for 10 min. Object exploration was defined as either nose touching the object or paw being in closed proximity (< 2 cm) to the object. The percentage of novel object preference was calculated as: novel object exploration time/ (novel object exploration time + familiar object exploration time) × 100%.

### Rota‐Rod Test

The rota‐rod test was used to examine the motor ability and balance of mice. The procedure was performed as previously described with modification.^[^ ^25^ ^]^ After training, the mice were placed on the rod, and the speed of the rotating rod was gradually increased from 4 to 40 rpm within 5 min. Each mouse performed five trials and the results were averaged. The length of time that mice remained on the rota‐rod was recorded and taken as the index of the rotarod test.

### Grip strength Test

A grip strength test was used to measure the forelimb grip strength by using the grip strength meter (BioSEB GS3). Mice were grabbed by the base of their tails and allowed them to freely grasp a grid connected to a force sensor with their front paws. The average of five consecutive trials was recorded.

### Forced Swimming Test

A forced swimming test was performed as previously described.^[^ ^73^ ^]^ Mice were placed in a clear cylinder filled with water (23 ± 2 °C) for 6 min. Immobility time in the total 6 min was measured.

### Statistical Analysis

All Results were present as Mean ± SEM. No statistical methods were used to predetermine sample sizes. The sample sizes (n) were provided in the figures and figure legends. All statistical analyses were performed using GraphPad Prism 6.0. For statistical analysis, unpaired two‐tailed Student's *t*‐test between two experimental groups or one‐way ANOVA with Dunnett's multiple‐comparison correction among three groups were applied. ^*^ *P* < 0.05, ^* *^ *P* < 0.01, ^* * *^ *P* < 0.001, or n.s., not significant.

## Conflict of Interest

The authors declare no conflict of interest.

## Author Contributions

W.W. and Y.W. contributed equally to this work. W.W. and J.J. designed the research; W.W. performed the research, and experiments, and drafted the manuscript and data analyses. Y W. contributed to image analysis, interpreted the data, and performed some experiments. L.S. and M.Z. provided important materials. T.Z. performed some experiments. J.Z., H.M., R.J., and D.Z. took care of mice and performed genotyping. L.C., Y.X., F.J., and H.L. provided some advice about experiences. J.J. supervised the project and acquired the funding support.

## Supporting information

Supporting Information

## Data Availability

The data that support the findings of this study are available from the corresponding author upon reasonable request.
