# Supplementary material for: Endothelial Cells Mediated by STING Regulate Oligodendrogenesis and Myelination During Brain Development
Source: Adv Sci (Weinh). 2024 Aug 13;11(38):2308508. doi: 10.1002/advs.202308508 (PMC11481185; doi:10.1002/advs.202308508)
Supplement: Supplementary file 1 — Supporting Information [file ADVS-11-2308508-s001.pdf]

ADVANCED SCIENCE
Open Access

Supporting Information

for *Adv. Sci.*, DOI 10.1002/advs.202308508

Endothelial Cells Mediated by STING Regulate Oligodendrogenesis and Myelination During Brain Development

Wenwen Wang, Yanyan Wang, Libo Su, Mengtian Zhang, Tianyu Zhang, Jinyue Zhao, Hongyan Ma, Dongming Zhang, Fen Ji, Ryan Dingli Jiao, Hong Li, Yuming Xu, Lei Chen* and Jianwei Jiao**

Supporting Information

Endothelial Cells Mediated by STING Regulate Oligodendrogenesis and Myelination During Brain Development

Wenwen Wang[1,2,9], Yanyan Wang[1,3,9], Libo Su[1,3], Mengtian Zhang[1,3], Tianyu Zhang[1,3], Jinyue Zhao[1,3], Hongyan Ma[1,3], Dongming Zhang[1,3], Fen Ji[1,3], Ryan Dingli Jiao[4], Hong Li[1,3], Yuming Xu[5*], Lei Chen[6*] and Jianwei Jiao[1,3,7,8,10*]

Affiliations:

[1] Key Laboratory of Organ Regeneration and Reconstruction, Chinese Academy of Science, Beijing 100101, China.

[2]School of Life Sciences, University of Science and Technology of China, Hefei 230026, China.

[3]University of Chinese Academy of Sciences, Beijing 100049, China.

[4]Beijing Royal School, Beijing, 102209, China.

[5]Department of Neurology, The First Affiliated Hospital of Zhengzhou University, Zhengzhou, 450000, China.

[6]Department of Neurology, West China Hospital, Sichuan University, Chengdu, 610041, China.

[7]Co-Innovation Center of Neuroregeneration, Nantong University, Nantong, 226001, China.

[8] Beijing Institute for Stem Cell and Regenerative Medicine, Institute for Stem Cell and Regeneration, Chinese Academy of Sciences, Beijing, 100101, China.

[9]These authors contributed equally

[10]Lead Contact

*Correspondence: jwjiao@ioz.ac.cn, chenlei@wchscu.cn, xuyuming@zzu.edu.cn

TableS1: Primer sequences target FDFT1

Name	Sequence (5‘-3’)
FDFT1-2K-F	AGGTGGTGCTTCAGCCTTAT
FDFT1-2K-R	AAAGGCTACTTAGCGCTGCC
FDFT1-1K-F	ATCTCAGGACTGGGTCCTCC
FDFT1-1K-R	AACAGCAGTGGCAGGCTAAA
FDFT1-0.5K-F	CCTCTTAAGCCTGCTGCGTA
FDFT1-0.5K-R	AGCGGGGTGGTATTTGTTCA
FDFT1-CDS-F	ATCAGACCAGTCGCAGCTTT
FDFT1-CDS-R	GTATCCAGGGCTCGGAGAAC

TableS2: RT-PCR Primers

Name	Sequence (5’-3’)
STING-RT-F	AAATAACTGCCGCCTCATTG
STING-RT-R	TGGGAGAGGCTGATCCATAC
cGAS-RT-F	GCTCACCAAAGATGCACAGC
cGAS-RT-R	GGTCCCCTTACGACTTTCCG
β-actin-RT-F	GGCTGTATTCCCCTCCATCG
β-actin-RT-R	CCAGTTGGTAACAATGCCATGT
SOX10-RT-F	AACCACCCCAAAGACAGAGC
SOX10-RT-R	TTGGGTGGCAGGTATTGGTC
PDGFRα-RT-F	GGCAAAGAACAACCTCAGCG
PDGFRα-RT-R	GACTCGATAACCCTCCAGCG
Olig2-RT-F	GAAGATCAACAGCCGCGAAC
Olig2-RT-R	GTAGATCTCGCTCACCAGTCG
MBP-RT-F	TTCTTTAGCGGTGACAGGGG
MBP-RT-R	GTTTTCATCTTGGGTCCGGC
PLP1-RT-F	GCCCTGACTGTTGTATGGCT
PLP1-RT-R	TACATTCTGGCATCAGCGCA
IL17D-RT-F	GAGGAGGACTTCCGCTTTCG
IL17D-RT-R	TGGTGATGTAGTGTTCGGCG
FDFT1-RT-F	ACGTCCTCACCTACCTGTCA

FDFT1-RT-R	ATAGCTTTGACGGCAGGCAT
CD31-RT-F	TGGAAGTGTCCTCCCTTGAGC
CD31-RT-R	AAGGGAGCCTTCCGTTCTTAGG
CDH5-RT-F	ATTGGCCTGTGTTTTCGCAC
CDH5-RT-R	CACAGTGGGGTCATCTGCAT
TNFα-RT-F	TGGAACTGGCAGAAGAG
TNFα-RT-R	CCATAGAACTGATGAGAGG
IL-6-RT-F	TAGTCCTTCCTACCCCAATTTCC
IL-6-RT-R	TTGGTCCTTAGCCACTCCTTC
IFNβ-RT-F	CAGCTCCAAGAAAGGACGAAC
IFNβ-RT-R	GGCAGTGTAACTCTTCTGCAT
IFIT2-RT-F	GCTCTGGAAAAGGACCCGAA
IFIT2-RT-R	GCTTCAGTGCCAAGAGGACT
CCL2-RT-F	TTAAAAACCTGGATCGGAACCAA
CCL2-RT-R	GCATTAGCTTCAGATTTACGGGT
IL-12B-RT-F	TGGTTTGCCATCGTTTTGCTG
IL-12B-RT-R	ACAGGTGAGGTTCACTGTTTCT
Vldlr-RT-F	AACTGCCCTTCTCGAACCTG
Vldlr-RT-R	TCAACACAGTCTCGGATGCC
CLU-RT-F	GAGATTCAGAACGCCGTCCA
CLU-RT-R	TCCCTAGTGTCCTCCAGAGC
CAV1-RT-F	CATCTGGAAGGCCAGCTTCA
CAV1-RT-R	TGCAGGAAGGAGAGAATGGC
Abcd2-RT-F	GCTGCAGAAATGTTACAAGGC
Abcd2-RT-R	AGATCACCATCTGCAAAGCCC
Abca2-RT-F	AAGCTTTCTACACCGCAGCA
Abca2-RT-R	GTGACCGTGGAGTTGGCATA
Osbpl6-RT-F	GACCTTGACACGGAAGAGCA
Osbpl6-RT-R	TCGGCGACCTCACAATTTCA
Ttc39b-RT-F	GAGTGGAATTGGACGCGGA
Ttc39b-RT-R	CCACCTTGGCTGATGAACCT

Figure S1

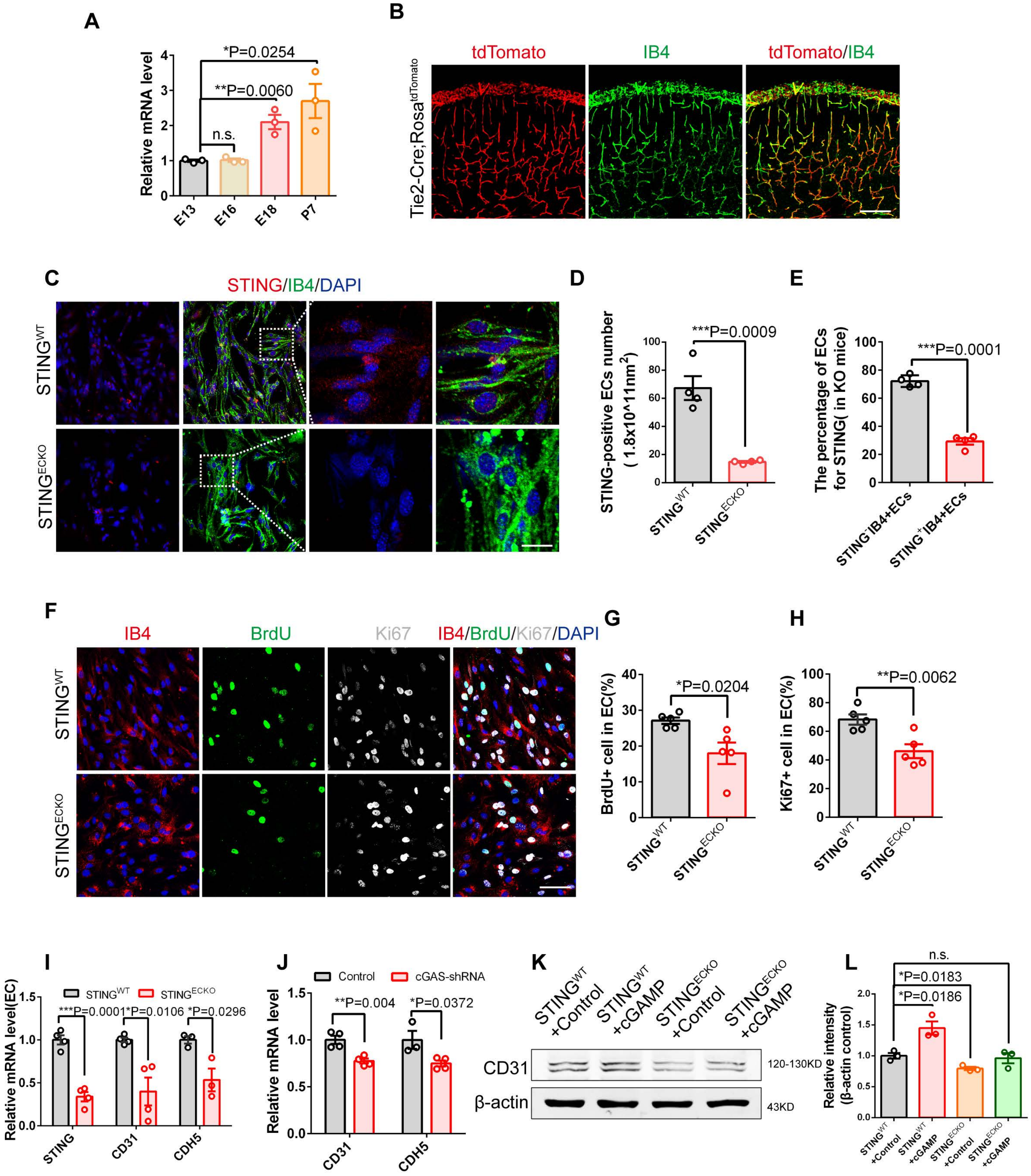

A
*P=0.0254
**P=0.0060
n.s.
Relative mRNA level
E13
E16
E18
P7
B
tdTomato
IB4
tdTomato/IB4
Tie2-Cre;Rosa tdTomato
C
STING/IB4/DAPI
STING WT
STING ECKO
D
***P=0.0009
STING-positive ECs number (1.8x10^11nm²)
E
***P=0.0001
The percentage of ECs for STING(in KO mice)
STING-IB4+ECs
STING+IB4+ECs
F
IB4
BrdU
Ki67
IB4/BrdU/Ki67/DAPI
G
*P=0.0204
BrdU+ cell in EC(%)
H
**P=0.0062
Ki67+ cell in EC(%)
I
***P=0.0001
*P=0.0106
*P=0.0296
Relative mRNA level(EC)
STING
CD31
CDH5
J
Control
cGAS-shRNA
**P=0.004
*P=0.0372
Relative mRNA level
K
STING WT +Control
STING WT +cGAMP
STING ECKO +Control
STING ECKO +cGAMP
CD31
β-actin
120-130KD
43KD
L
n.s.
*P=0.0183
*P=0.0186
Relative intensity (β-actin control)

Figure S1. STING signaling affects angiogenesis, Related to Figure 1.

(**A**)RT-PCR was performed to detect the mRNA levels of STING in endothelial cells at different developmental stages. n.s., not significant, *P<0.05, **P<0.01 (mean ± SEM, n = 3 mice each group).

(B) Confocal immunofluorescence image of tdTomato and IB4 in Tie2-Cre; Rosa26^{tdTomato} mice cortical region. Scale bar, 100μm.

(**C**) Confocal immunofluorescence image of IB4 and STING in the *STING*^{WT} and *STING*^{ECKO} isolated brain endothelial cells. Scale bar, 100μm.

(**D**) Quantification of STING positive cells numbers in the *STING*^{WT} and *STING*^{ECKO} isolated brain endothelial cells. ***P<0.001(mean ± SEM, unpaired two-tailed Student's *t* test, n =4 each group from 3 independent experiments).

(**E**) Quantification shows the percentage of EC cells negative and positive for STING in KO mice. ***P<0.001 (n =4 each group from 3 independent experiments)

(**F**) Confocal immunofluorescence image of IB4, BrdU and Ki67 at E18 in the *STING*^{WT} and *STING*^{ECKO} isolated brain endothelial cells. Scale bar, 50μm.

(**G**) Quantification showing the decreased number of BrdU^{+} endothelial cells in *STING*^{ECKO} mice. *P<0.05 (mean ± SEM, unpaired two-tailed Student's t test, n =5 mice each group).

(**H**) Quantification showing the decreased number of Ki67^{+} endothelial cells in *STING*^{ECKO} mice. **P<0.01 (mean ± SEM, unpaired two-tailed Student's t test, n =5 mice each group).

(**I**) RT-PCR analysis of the mRNA expression level of STING, CD31 and CDH5 at

E18 in isolated *STING*^{WT} and *STING*^{ECKO} mice brain ECs. *P<0.05, ***P<0.001 (mean ± SEM, unpaired two-tailed Student's *t* test, STING and CD31: n=4 mice each group; CDH5: n=3mice each group).

(**J**) RT-PCR analysis of the mRNA expression level of CD31 and CDH5 in ECs after knockdown of cGAS. *P<0.05, **P<0.01 (mean ± SEM, unpaired two-tailed Student's *t* test, CD31: n=4 sample each group; CDH5: Control n=3sample; cGAS-shRNA n=4sample).

(**K**) Western blot analysis of the expression level of CD31 at E18 in isolated ECs *STING*^{WT} and *STING*^{ECKO} mice after treating with exogenous cGAMP(2μM) for 1 day. β-actin was detected as loading control.

(**L**) Statistics of relative intensity of CD31 in *STING*^{WT} and *STING*^{ECKO} mice after treating with exogenous cGAMP. n.s., not significant, *P<0.05. (mean ± SEM, unpaired two-tailed Student's t test, n=3 independent experiments).

Data are represented as means ± SEM. unpaired two-tailed Student's *t* test. At least three biological replicates are shown. n.s., not significant, *P<0.05, **P<0.01, ***P<0.001.

FigureS2

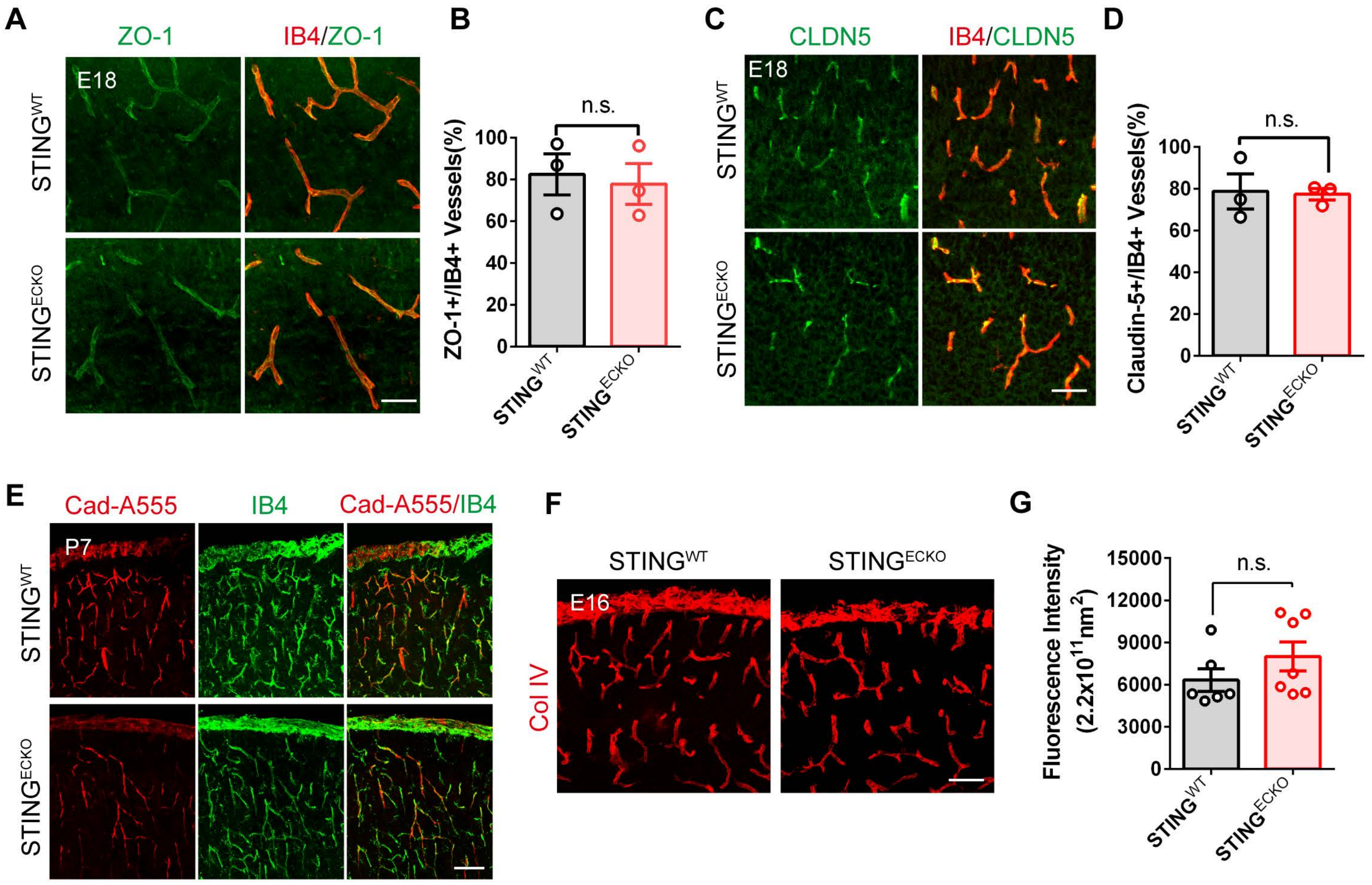
A
ZO-1
IB4/ZO-1
E18
STING^WT
STING^ECKO
B
n.s.
ZO-1+/IB4+ Vessels(%)
100
80
60
40
20
0
STING^WT
STING^ECKO
C
CLDN5
IB4/CLDN5
E18
STING^WT
STING^ECKO
D
n.s.
Claudin-5+/IB4+ Vessels(%)
100
80
60
40
20
0
STING^WT
STING^ECKO
E
Cad-A555
IB4
Cad-A555/IB4
P7
STING^WT
STING^ECKO
F
STING^WT
STING^ECKO
E16
Col IV
G
n.s.
Fluorescence Intensity
(2.2x10^11nm^2)
15000
12000
9000
6000
3000
0
STING^WT
STING^ECKO

Figure S2. Loss of STING in endothelial cells does not affect blood-brain barrier integrity, Related to Figure 1.

(**A**) Confocal immunofluorescence image of IB4 and ZO-1 at E18 in $STING^{WT}$ and $STING^{ECKO}$ mice. Scale bar, 50μm.

(**B**) Quantification of the percent of ZO-1^{+} IB4^{+} vessels showing no statistical difference between $STING^{WT}$ and $STING^{ECKO}$ mice. n.s., not significant. (mean ± SEM, unpaired two-tailed Student's *t* test, n =3 mice each group).

(**C**) Confocal immunofluorescence image of IB4 and Claudin-5 at E18 in $STING^{WT}$ and $STING^{ECKO}$ mice. Scale bar, 50μm.

(**D**) Quantification of the percent of Claudin-5^{+} IB4^{+} vessels showing no statistical difference between $STING^{WT}$ and $STING^{ECKO}$ mice. n.s., not significant. (mean ± SEM, unpaired two-tailed Student's *t* test, n =3 mice each group).

(**E**) Confocal immunofluorescence image of IB4 and Cad-A555 showing no cadaverine extravasation at P7 in $STING^{WT}$ and $STING^{ECKO}$ mice from the blood vessels. Scale bar, 100μm.

(**F**) Confocal immunofluorescence image of Col IV at E16 in $STING^{WT}$ and $STING^{ECKO}$ mice brain cortex. Scale bar, 100μm.

(**G**) Quantification of the fluorescence intensity of Col IV staining showing no statistical difference between $STING^{WT}$ and $STING^{ECKO}$ mice. n.s., not significant. (mean ± SEM, unpaired two-tailed Student's *t* test, $STING^{WT}$ n =6 mice; $STING^{ECKO}$ n =7 mice).

Data are represented as means ± SEM. unpaired two-tailed Student's *t* test. At least

three biological replicates are shown. n.s., not significant.

Figure S3

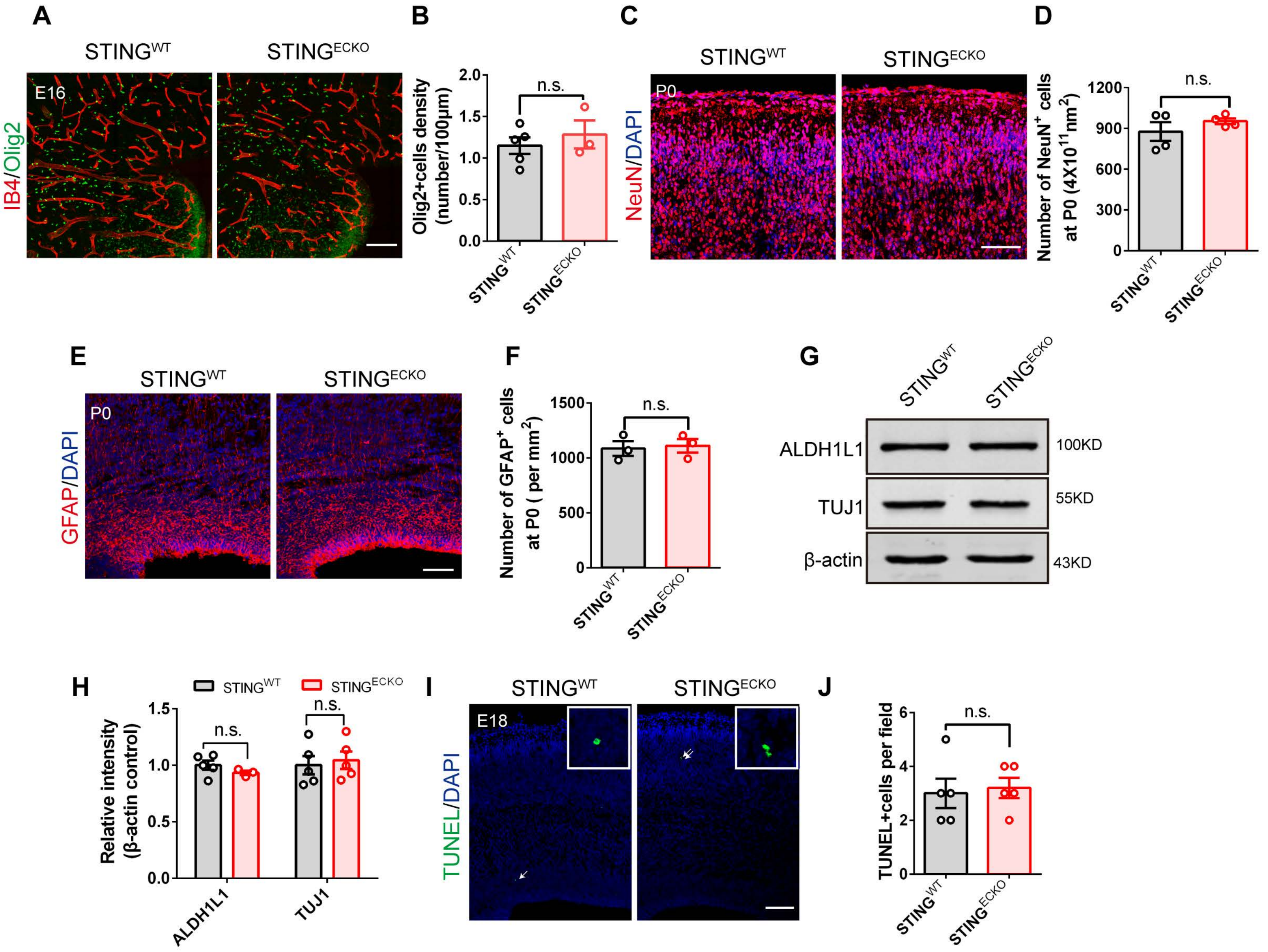
A
STING^WT
STING^ECKO
E16
IB4/Olig2
B
Olig2+cells density (number/100μm)
n.s.
STING^WT
STING^ECKO
C
STING^WT
STING^ECKO
P0
NeuN/DAPI
D
Number of NeuN+ cells at P0 (4X10^11nm^2)
n.s.
E
STING^WT
STING^ECKO
P0
GFAP/DAPI
F
Number of GFAP+ cells at P0 (per mm^2)
n.s.
G
ALDH1L1
TUJ1
β-actin
100KD
55KD
43KD
H
Relative intensity (β-actin control)
n.s.
ALDH1L1
TUJ1
I
E18
TUNEL/DAPI
J
TUNEL+cells per field
n.s.

Figure S3. Endothelial STING deletion does not affect neurons and astrocytes production, Related to Figure 2.

(**A**) Confocal immunofluorescence image of IB4 and Olig2 at E16 in $STING^{WT}$ and $STING^{ECKO}$ mice. Scale bars, 100μm (left), 10μm (right).

(**B**) Quantification of the number of Olig2^{+} OPCs on a vessel in $STING^{WT}$ and $STING^{ECKO}$ mice, expressed as number of cells on each 100μm IB4 vessel. n.s., not significant. (mean ± SEM, unpaired two-tailed Student's *t* test, $STING^{WT}$ n =5 mice; $STING^{ECKO}$ n =3 mice).

(**C**) Confocal immunofluorescence image of NeuN at P0 in $STING^{WT}$ and $STING^{ECKO}$ mice cortical region. Scale bars, 50μm.

(**D**) Quantification of the number of NeuN^{+} neurons showing no statistical difference between $STING^{WT}$ and $STING^{ECKO}$ mice cortical region. n.s., not significant. (mean ± SEM, unpaired two-tailed Student's *t* test, n =4 mice each group).

(**E**) Confocal immunofluorescence image of GFAP at P0 in $STING^{WT}$ and $STING^{ECKO}$ mice. Scale bars, 100μm.

(**F**) Quantification of the number of GFAP^{+} astrocytes showing no statistical difference between $STING^{WT}$ and $STING^{ECKO}$ mice. n.s., not significant (mean ± SEM, unpaired two-tailed Student's *t* test, n =3 mice each group).

(**G**) Western blot analysis of the expression levels of astrocyte marker ALDH1L1and neuron marker TUJ1 at P0 in $STING^{WT}$ and $STING^{ECKO}$ mice brain cortex. β-actin was detected as loading control.

(**H**) Statistics of relative intensity of TUJ1and GFAP showing no changes in the

expression of TUJ1$^+$ cells and the expression ALDH1L1$^+$ cells between *STING*^{WT} and *STING*^{ECKO} mice. n.s., not significant (mean ± SEM, unpaired two-tailed Student's *t* test, ALDH1L1: *STING*^{WT} n =5 mice; *STING*^{ECKO} n =3 mice TUJ1: n=5 mice each group).

(**I**) Confocal immunofluorescence image of TUNEL$^+$ cells at E18 in *STING*^{WT} and *STING*^{ECKO} mice brain cortex. The white box represents the enlarged image at the arrow. Scale bar, 100µm.

(**J**) Quantification of the number of TUNEL$^+$ cells showing no statistical difference between *STING*^{WT} and *STING*^{ECKO} mice. n.s., not significant (mean ± SEM, unpaired two-tailed Student's *t* test, n =5 mice each group).

Data are represented as means ± SEM. unpaired two-tailed Student's *t* test. At least three biological replicates are shown. n.s., not significant.

Figure S4

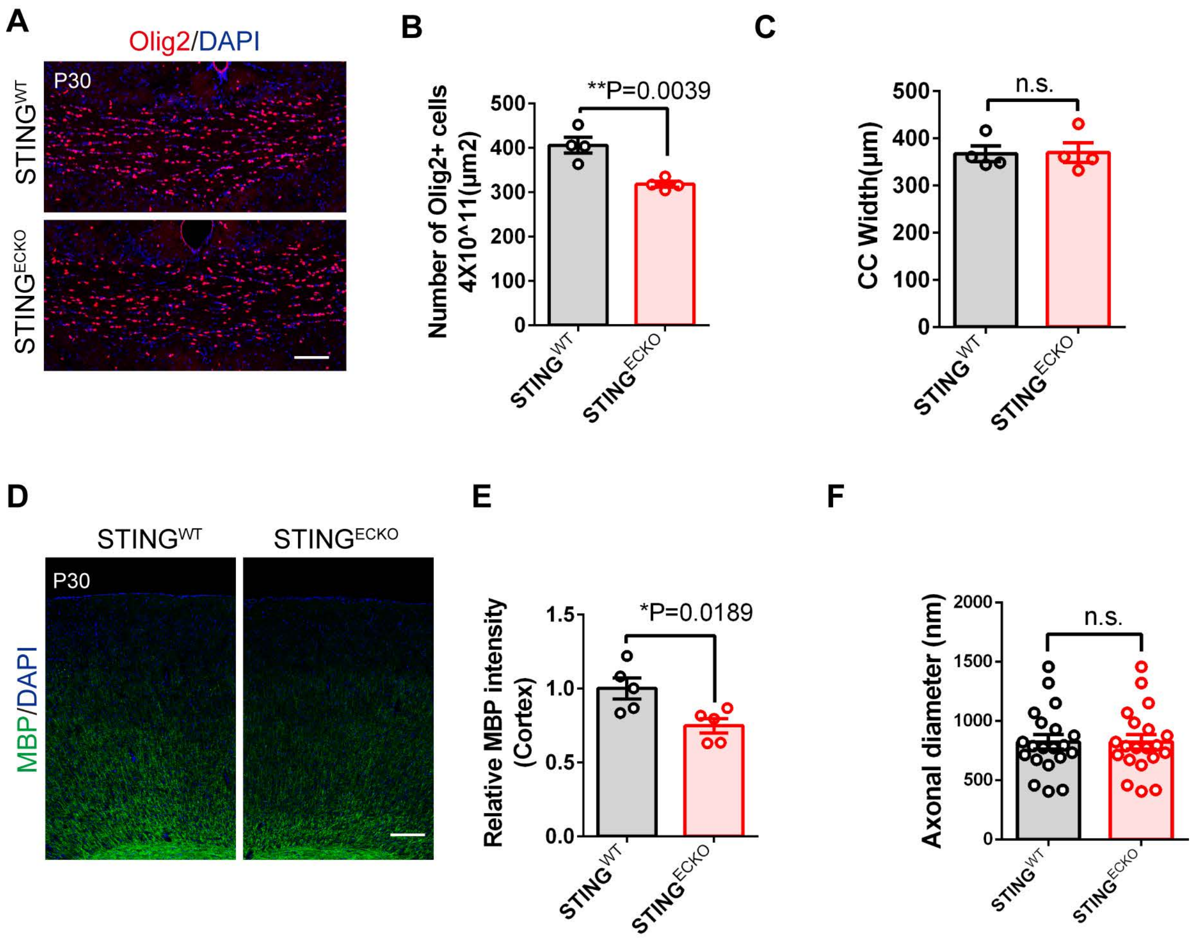
A
Olig2/DAPI
P30
STING^WT
STING^ECKO
B
Number of Olig2+ cells 4X10^11(μm2)
**P=0.0039
500
400
300
200
100
0
STING^WT
STING^ECKO
C
CC Width(μm)
n.s.
500
400
300
200
100
0
STING^WT
STING^ECKO
D
STING^WT
STING^ECKO
P30
MBP/DAPI
E
Relative MBP intensity (Cortex)
*P=0.0189
1.5
1.0
0.5
0.0
STING^WT
STING^ECKO
F
Axonal diameter (nm)
n.s.
2000
1500
1000
500
0
STING^WT
STING^ECKO

Figure S4. Endothelial STING deletion affects oligodendrocyte production, Related to Figure 3.

(**A**) Confocal immunofluorescence image of $Olig2^+$ cells at P30 in the *$STING^{WT}$* and *$STING^{ECKO}$* corpus callosum. Scale bar, 100μm.

(**B**) Quantification showing that the number of $Olig2^+$ cells was decreased in the *$STING^{WT}$* and *$STING^{ECKO}$* corpus callosum. **P<0.01 (mean ± SEM, unpaired two-tailed Student's *t* test, n=4 mice each group).

(**C**) Quantification showed corpus callosum width at the midline of *$STING^{WT}$* and *$STING^{ECKO}$* mice. n.s., not significant (mean ± SEM, unpaired two-tailed Student's *t* test, n=4 mice each group).

(**D**) Confocal immunofluorescence image of MBP^+ cells at P30 in the *$STING^{WT}$* and *$STING^{ECKO}$* cerebral cortex. Scale bar, 200μm.

(**E**) Relative quantification of MBP fluorescence intensity was decreased in the *$STING^{WT}$* and *$STING^{ECKO}$* cerebral cortex. *P<0.05 (mean ± SEM, unpaired two-tailed Student's *t* test, n=5 mice each group).

(**F**) Quantification of axonal diameter showing no statistical difference between *$STING^{WT}$* and *$STING^{ECKO}$* mice. n.s., not significant. (mean ± SEM, unpaired two-tailed Student's *t* test, n=20 each group from 3 independent experiments).

Data are represented as means ± SEM. unpaired two-tailed Student's *t* test. At least three biological replicates are shown. n.s., not significant, *P<0.05, **P<0.01.

Figure S5

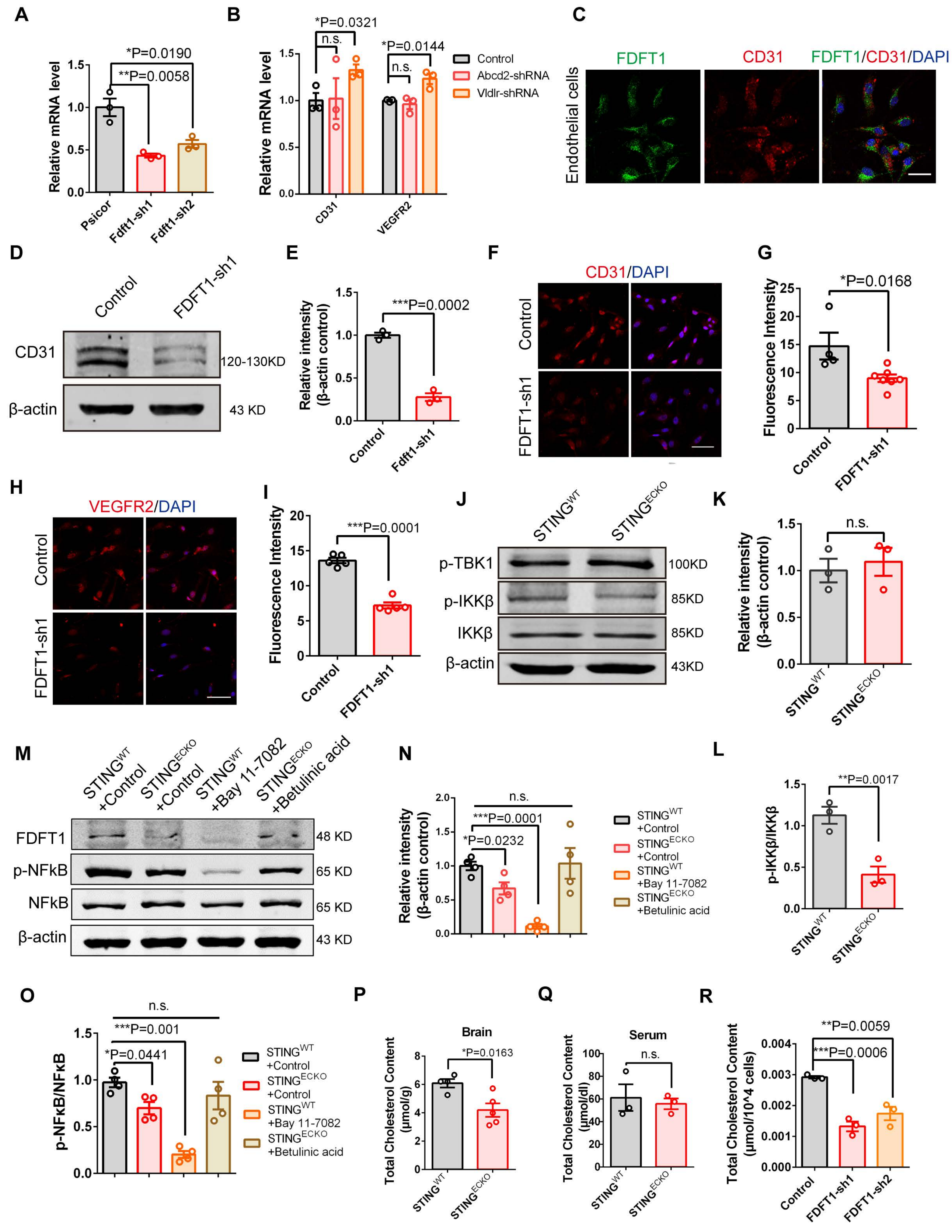
A
Relative mRNA level
*P=0.0190
**P=0.0058
Psicor
Fdft1-sh1
Fdft1-sh2
B
Relative mRNA level
*P=0.0321
n.s.
*P=0.0144
n.s.
Control
Abcd2-shRNA
Vldlr-shRNA
CD31
VEGFR2
C
FDFT1
CD31
FDFT1/CD31/DAPI
Endothelial cells
D
Control
FDFT1-sh1
CD31
120-130KD
β-actin
43 KD
E
Relative intensity (β-actin control)
***P=0.0002
Control
Fdft1-sh1
F
CD31/DAPI
Control
FDFT1-sh1
G
Fluorescence Intensity
*P=0.0168
Control
FDFT1-sh1
H
VEGFR2/DAPI
Control
FDFT1-sh1
I
Fluorescence Intensity
***P=0.0001
Control
FDFT1-sh1
J
STING^WT
STING^ECKO
p-TBK1
100KD
p-IKKβ
85KD
IKKβ
85KD
β-actin
43KD
K
Relative intensity (β-actin control)
n.s.
STING^WT
STING^ECKO
M
STING^WT +Control
STING^ECKO +Control
STING^WT +Bay 11-7082
STING^ECKO +Betulinic acid
FDFT1
48 KD
p-NFkB
65 KD
NFkB
65 KD
β-actin
43 KD
N
Relative intensity (β-actin control)
n.s.
***P=0.0001
*P=0.0232
STING^WT +Control
STING^ECKO +Control
STING^WT +Bay 11-7082
STING^ECKO +Betulinic acid
L
p-IKKβ/IKKβ
**P=0.0017
STING^WT
STING^ECKO
O
p-NFκB/NFκB
n.s.
***P=0.001
*P=0.0441
STING^WT +Control
STING^ECKO +Control
STING^WT +Bay 11-7082
STING^ECKO +Betulinic acid
P
Brain
Total Cholesterol Content (μmol/g)
*P=0.0163
STING^WT
STING^ECKO
Q
Serum
Total Cholesterol Content (μmol/dl)
n.s.
STING^WT
STING^ECKO
R
Total Cholesterol Content (μmol/10^4 cells)
**P=0.0059
***P=0.0006
Control
FDFT1-sh1
FDFT1-sh2

Figure S5. FDFT1 is downstream target of STING in endothelial cells, Related to Figure 5.

(**A**) RT-PCR analysis showing the knockdown efficiency of FDFT1-shRNA at the mRNA level. *P<0.05, **P<0.01(mean ± SEM, unpaired two-tailed Student's *t* test, n = 3 independent experiments).

(**B**) RT-PCR analysis showing the mRNA expression level of CD31 and VEGFR2 was reduced after knockdown of ABCD2 and VLDLR in primary ECs. n.s., not significant, *P<0.05. (mean ± SEM, unpaired two-tailed Student's *t* test, n = 3 independent experiments).

(**C**) Confocal immunofluorescence image of FDFT1 and CD31 showing that FDFT1 was expressed in primary endothelial cells. Scale bar, 20μm.

(**D**) Western blot analysis of the expression levels of CD31in primary endothelial cells after knockdown FDFT1. β-actin was detected as loading control.

(**E**) Statistics of relative intensity of CD31 showed that CD31 expression in endothelial cells decreased after knock down of FDFT1. ***P<0.001 (mean ± SEM, unpaired two-tailed Student's *t* test, n=3 independent experiments).

(**F**) Confocal immunofluorescence image of $CD31^+$ cells in primary EC. Scale bar, 20μm.

(**G**) CD31 fluorescence intensity was decreased after knockdown of FDFT1. *P<0.05 (mean ± SEM, unpaired two-tailed Student's *t* test, Control: n=4; FDFT1-sh1: n= 7 from 3 independent experiments).

(**H**) Confocal immunofluorescence image of $VEGFR2^+$ cells in primary EC. Scale bar,

20μm.

(**I**) VEGFR2 fluorescence intensity was decreased after knock down of FDFT1. ***P<0.0001 (mean ± SEM, unpaired two-tailed Student's *t* test, n=5 sample from 3 independent experiments).

(**J**) Western blot analysis of the expression levels of p-TBK1, p-IKKβ and IKKβ in $STING^{WT}$ and $STING^{ECKO}$ mice endothelial cells. β-actin was detected as loading control.

(**K**) Statistics of relative intensity of p-TBK1showed no changes between $STING^{WT}$ and $STING^{ECKO}$ mice. n.s., not significant (mean ± SEM, unpaired two-tailed Student's *t* test n=3 mice each group).

(**L**) Statistics showing the decreased ratio of p-IKKβ and IKKβ in $STING^{ECKO}$ brain endothelial cells. **P<0.01 (mean ± SEM, unpaired two-tailed Student's *t* test, n =3 mice each group).

(**M**) Western blot analysis of FDFT1 and p-NFκB and NFκB treated with 10μM BAY11-7085NF-κB inhibitor) and 10μM Betulinic acid (NF-κB activator). β-actin was detected as loading control.

(**N** and **O**) Statistics of relative intensity of FDFT1 and the ratio of p-NF*κ*B and NF*κ*B. n.s., not significant, *P<0.05, ***P<0.001 (mean ± SEM, unpaired two-tailed Student's t test, n=4 each group)

(**P**) Statistics showing cholesterol levels were reduced in $STING^{ECKO}$ mice brain. *P<0.05 (mean ± SEM, unpaired two-tailed Student's *t* test, $STING^{WT}$ n=4mice; $STING^{ECKO}$ n=5mice).

(**Q**) Statistics showing cholesterol levels were no statistical difference between *STING*^{WT} and *STING*^{ECKO} mice serum. n.s., not significant (mean ± SEM, unpaired two-tailed Student's *t* test, n=3mice each group).

(**R**) Statistics showing cholesterol levels were reduced after knockdown of FDFT1in endothelial cells. **P<0.01,***P<0.001 (mean ± SEM, unpaired two-tailed Student's *t* test, n=3 independent experiments).

Data are represented as means ± SEM. unpaired two-tailed Student's *t* test. At least three biological replicates are shown. n.s., not significant, *P<0.05, **P<0.01, ***P<0.01.

Figure S6

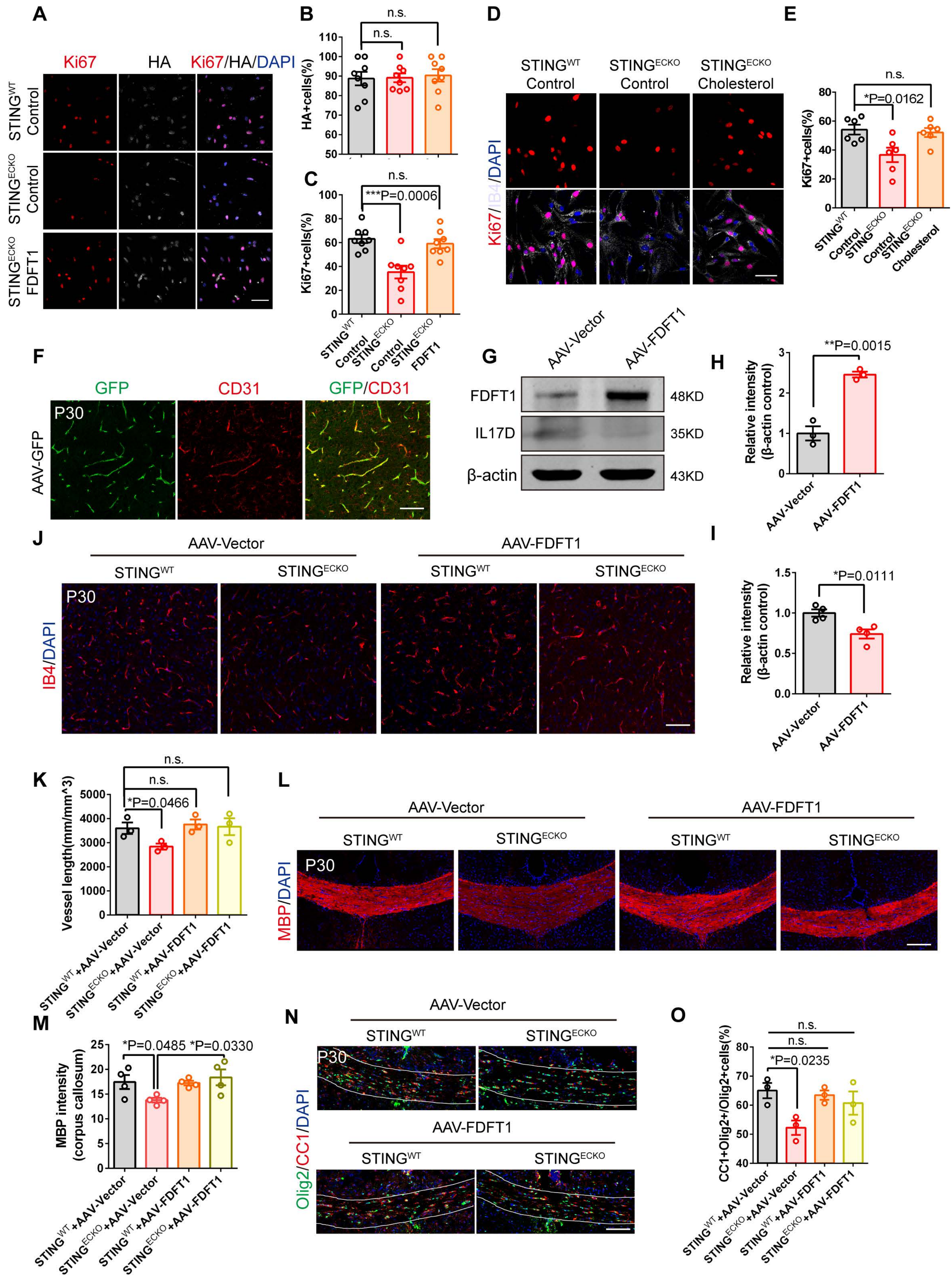

A
Ki67
HA
Ki67/HA/DAPI
STING^WT Control
STING^ECKO Control
STING^ECKO FDFT1
B
n.s.
n.s.
HA+cells(%)
C
n.s.
***P=0.0006
Ki67+cells(%)
STING^WT Control
STING^ECKO Control
STING^ECKO FDFT1
D
STING^WT Control
STING^ECKO Control
STING^ECKO Cholesterol
Ki67/IB4/DAPI
E
n.s.
*P=0.0162
Ki67+cells(%)
STING^WT Control
STING^ECKO Control
STING^ECKO Cholesterol
F
GFP
CD31
GFP/CD31
P30
AAV-GFP
G
AAV-Vector
AAV-FDFT1
FDFT1
48KD
IL17D
35KD
β-actin
43KD
H
**P=0.0015
Relative intensity (β-actin control)
AAV-Vector
AAV-FDFT1
I
*P=0.0111
Relative intensity (β-actin control)
AAV-Vector
AAV-FDFT1
J
AAV-Vector
AAV-FDFT1
STING^WT
STING^ECKO
STING^WT
STING^ECKO
P30
IB4/DAPI
K
n.s.
n.s.
*P=0.0466
Vessel length(mm/mm^3)
STING^WT+AAV-Vector
STING^ECKO+AAV-Vector
STING^WT+AAV-FDFT1
STING^ECKO+AAV-FDFT1
L
AAV-Vector
AAV-FDFT1
STING^WT
STING^ECKO
STING^WT
STING^ECKO
P30
MBP/DAPI
M
*P=0.0485
*P=0.0330
MBP intensity (corpus callosum)
STING^WT+AAV-Vector
STING^ECKO+AAV-Vector
STING^WT+AAV-FDFT1
STING^ECKO+AAV-FDFT1
N
AAV-Vector
STING^WT
STING^ECKO
P30
AAV-FDFT1
STING^WT
STING^ECKO
Olig2/CC1/DAPI
O
n.s.
n.s.
*P=0.0235
CC1+Olig2+/Olig2+cells(%)
STING^WT+AAV-Vector
STING^ECKO+AAV-Vector
STING^WT+AAV-FDFT1
STING^ECKO+AAV-FDFT1

Figure S6. Overexpression of FDFT1 rescues impaired angiogenesis and Oligodendrogenesis in *STING*^{ECKO} mice. Related to Figure 5.

(**A**) Confocal immunofluorescence image showed that FDFT1 can rescue damaged angiogenesis caused by endothelial STING deletion. Scale bar, 100μm.

(**B**) Quantification of the percent of HA^{+} cells showing transfection efficiency of FDFT1.

(**C**) Quantification of the percent of $Ki67^{+}$ cells. ***P<0.001 (mean ± SEM, unpaired two-tailed Student's *t* test, n =8 from 3 independent experiments).

(**D**) Confocal immunofluorescence image showed that exogenous cholesterol can rescue endothelial cell proliferation caused by endothelial STING deletion. Scale bar, 100μm.

(**E**) Quantification of the percent of $Ki67^{+}$cells. *P<0.01 (mean ± SEM, unpaired two-tailed Student's t test, each group n =6 from 3 independent experiments).

(**F**) Confocal images of immunostaining of GFP for AAV transduced ECs (green), and CD31 for ECs (red) showing effective AAV-mediated GFP expression in brain ECs. Scale bar,50 µm.

(**G**) Western blot analysis showing the expression level of FDFT1 and IL17D in ECs afterAAV-FDFT1 injection. β-actin was detected as loading control.

(**H**) Statistics of relative intensity of FDFT1 showing the increased expression of FDFT1 in isolated brain endothelial cells afterAAV-FDFT1 injection. **P<0.005 (mean ± SEM, unpaired two-tailed Student's t test, n=3 mice each group).

(**I**) Statistics of relative intensity of IL17D showing the decreased expression of IL17D in isolated brain endothelial cells afterAAV-FDFT1 injection. *P<0.05 (mean

± SEM, unpaired two-tailed Student's t test, n=4 mice each group).

(**J**) Confocal immunofluorescence image of IB4 of *STING*^{WT} and *STING*^{ECKO} mice treated with AAV-Vector or AAV-FDFT1.Scale bar, 50μm.

(**K**) Quantification of the vessel length and showing that AAV-based overexpression of FDFT1 in the endothelial cells rescued aberrant angiogenesis in *STING*^{ECKO} mice. *P<0.05 (mean ± SEM, unpaired two-tailed Student's t test, n=3 mice each group).

(**L**) Confocal immunofluorescence image of MBP of *STING*^{WT} and *STING*^{ECKO} mice treated with AAV-Vector or AAV-FDFT1.Scale bar, 200μm.

(**M**) Quantification of MBP fluorescence intensity and showing that AAV-based overexpression of FDFT1 in the endothelial cells rescued aberrant oligodendrogenesis in *STING*^{ECKO} mice at P30. *P<0.05 (mean ± SEM, unpaired two-tailed Student's t test, n=4 mice each group).

(**N**) Confocal immunofluorescence image of CC1 and Olig2 of *STING*^{WT} and *STING*^{ECKO} mice treated with AAV-Vector or AAV-FDFT1.Scale bar, 100μm.

(**O**) Quantification of the percent of $CC1^+$ cells among Olig2 cells in *STING*^{WT} and *STING*^{ECKO} mice treated with AAV-Vector or AAV-FDFT1. *P<0.05 (mean ± SEM, unpaired two-tailed Student's t test, n=3 mice each group).

Data are represented as means ± SEM. unpaired two-tailed Student's *t* test. At least three biological replicates are shown. n.s., not significant, *P<0.05, **P<0.01, ***P<0.01.

Figure S7

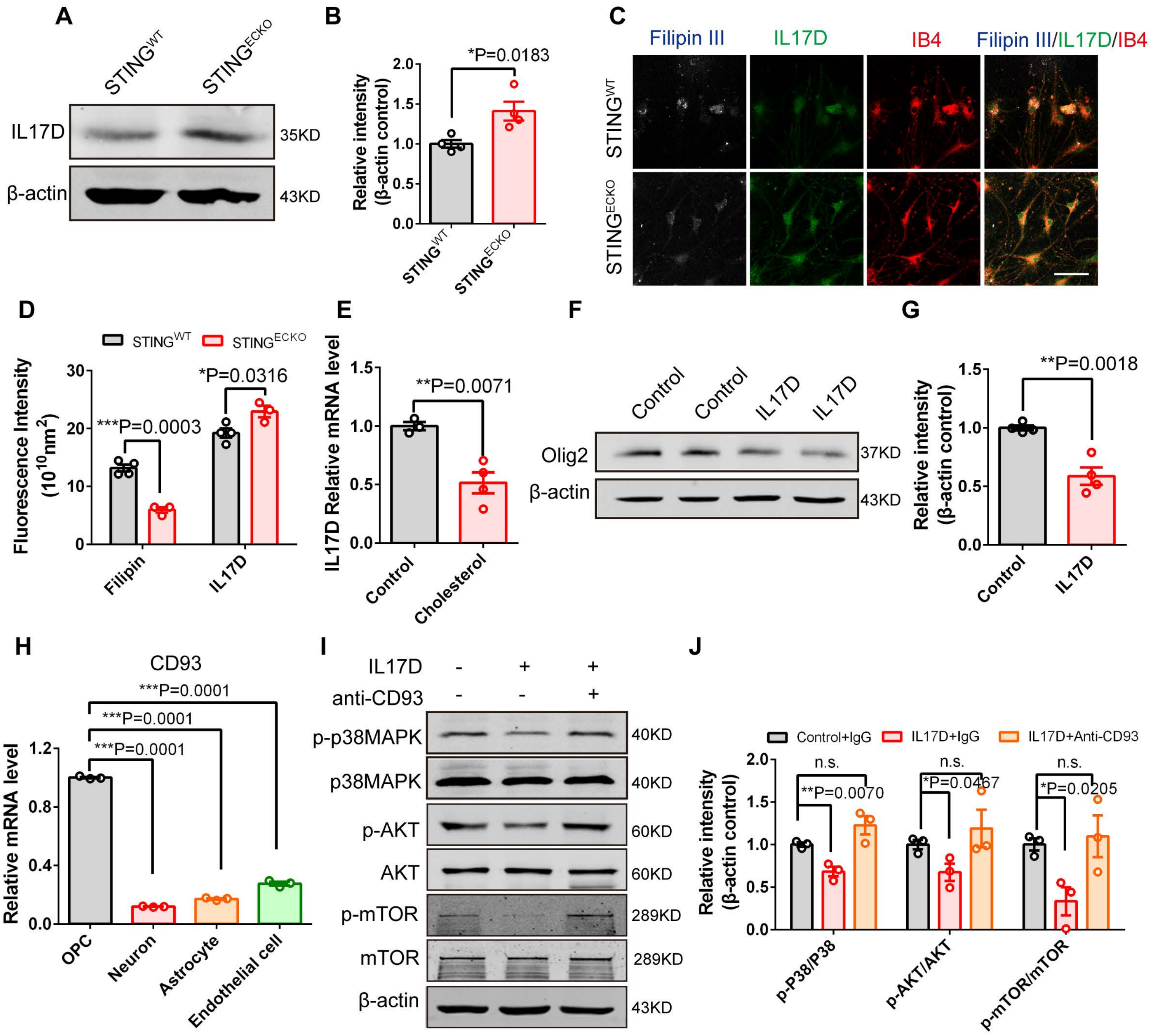

A
STING^WT
STING^ECKO
IL17D
35KD
β-actin
43KD
B
*P=0.0183
Relative intensity (β-actin control)
C
Filipin III
IL17D
IB4
Filipin III/IL17D/IB4
D
STING^WT
STING^ECKO
Fluorescence Intensity (10^10nm^2)
***P=0.0003
*P=0.0316
Filipin
IL17D
E
IL17D Relative mRNA level
**P=0.0071
Control
Cholesterol
F
Control
IL17D
Olig2
37KD
β-actin
43KD
G
**P=0.0018
Relative intensity (β-actin control)
Control
IL17D
H
CD93
***P=0.0001
Relative mRNA level
OPC
Neuron
Astrocyte
Endothelial cell
I
IL17D
anti-CD93
p-p38MAPK
40KD
p38MAPK
40KD
p-AKT
60KD
AKT
60KD
p-mTOR
289KD
mTOR
289KD
β-actin
43KD
J
Control+IgG
IL17D+IgG
IL17D+Anti-CD93
Relative intensity (β-actin control)
n.s.
**P=0.0070
*P=0.0467
*P=0.0205
p-P38/P38
p-AKT/AKT
p-mTOR/mTOR

Figure S7. IL17D inhibits oligodendrogenesis, Related to Figure 6.

(**A**) Western blot analysis of the expression levels of IL17D in *STING*^{WT} and *STING*^{ECKO} mice endothelial cells. β-actin was detected as loading control.

(**B**) Statistics of relative intensity of IL17D showing the increased expression of IL17D in *STING*^{ECKO} isolated brain endothelial cells. *P<0.05 (mean ± SEM, unpaired two-tailed Student's *t* test, n=4 mice each group).

(**C**) Confocal immunofluorescence image of Filipin, IL17D and IB4 showing that cholesterol production decreased and IL17D increasing in *STING*^{ECKO} endothelial cells. Scale bar, 50μm.

(**D**) Quantification of fluorescence intensity of Filipin and IL17D in *STING*^{WT} and *STING*^{ECKO} mice endothelial cells. *P<0.05, ***P<0.001 (mean ± SEM, unpaired two-tailed Student's *t* test, *STING*^{WT}: n=4 mice; *STING*^{ECKO}: n=3 mice).

(**E**) RT-PCR analysis of the mRNA expression level of IL17D after adding cholesterol into endothelial cells. **P<0.01 (mean ± SEM, unpaired two-tailed Student's *t* test, Control: n=3 sample; cholesterol: n=4 sample).

(**F**) Western blot analysis of the expression levels of Olig2 after adding IL17D 2day in oligodendrocyte precursor cells. β-actin was detected as loading control.

(**G**) Statistics of relative intensity of Olig2 showing the decreased expression of OLIG2 after adding IL17D 2day in OPCs. **P<0.01 (mean ± SEM, unpaired two-tailed Student's *t* test, n=4 each group).

(**H**) RT-PCR analysis of the mRNA expression level of CD93 in primary OPC, neuron, astrocyte and endothelial cells. ***P<0.001 (mean ± SEM, unpaired two-tailed

Student's *t* test, n=3 independent experiments).

(**I**) Western blot analysis of the expression levels of p-P38MAPK, P38MAPK, AKT, p-AKT, mTOR and p-mTOR after exogenous IL17D treatment of OPC blocking CD93. β-actin was detected as loading control.

(**J**) Statistics showing the decreased ratio of p-P38MAPK/P38MAPK, p-AKT /AKT and p-mTOR /mTOR after adding IL17D in OPCs. After blocking CD93 function, a rescue of the phenotype was characterized by reduced expression levels of p-P38MAPK, p-AKT, and p-mTOR. n.s., not significant, *P<0.05, **P<0.01 (mean ± SEM, unpaired two-tailed Student's *t* test, n =3 independent experiments).

Data are represented as means ± SEM. unpaired two-tailed Student's *t* test. At least three biological replicates are shown. n.s., not significant, *P<0.05, **P<0.01, ***P<0.001.

Figure S8

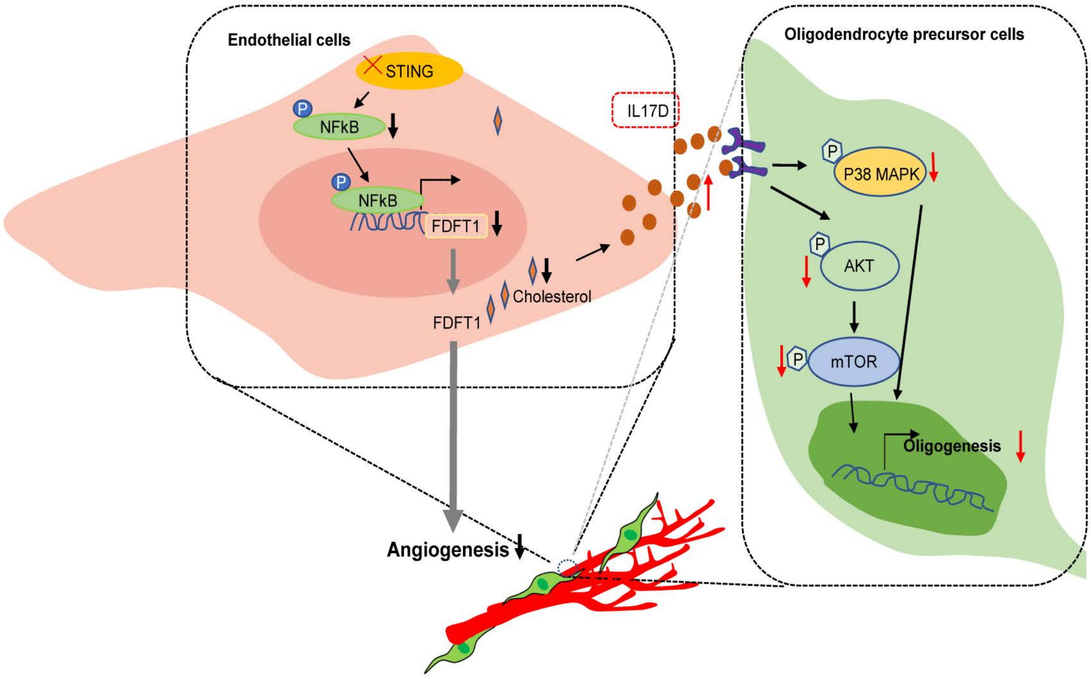

Endothelial cells
STING
P
NFkB
P
NFkB
FDFT1
Cholesterol
FDFT1
IL17D
Angiogenesis
Oligodendrocyte precursor cells
P
P38 MAPK
P
AKT
P
mTOR
Oligogenesis

Figure S8. The summary models

Model showing how endothelial STING regulates regulate oligodendrogenesis and myelination during brain development
